# Indicators for evaluating European population health: a Delphi selection process

**DOI:** 10.1186/s12889-018-5463-0

**Published:** 2018-04-27

**Authors:** Ângela Freitas, Paula Santana, Mónica D. Oliveira, Ricardo Almendra, João C. Bana e Costa, Carlos A. Bana e Costa

**Affiliations:** 1 Centre of Studies in Geography and Spatial Planning (CEGOT), University of Coimbra, Coimbra, Portugal; 2 Department of Geography and Tourism, Centre of Studies in Geography and Spatial Planning (CEGOT), University of Coimbra, Coimbra, Portugal; 3 Centre for Management Studies of Instituto Superior Técnico (CEG-IST), Universidade de Lisboa, Lisbon, Portugal; 4 Bana Consulting, Lda, Lisbon, Portugal

**Keywords:** Population health, European regions, Indicators, Participatory approach, Web Delphi, Expert opinion, Group agreement

## Abstract

**Background:**

Indicators are essential instruments for monitoring and evaluating population health. The selection of a multidimensional set of indicators should not only reflect the scientific evidence on health outcomes and health determinants, but also the views of health experts and stakeholders. The aim of this study is to describe the Delphi selection process designed to promote agreement on indicators considered relevant to evaluate population health at the European regional level.

**Methods:**

Indicators were selected in a Delphi survey conducted using a web-platform designed to implement and monitor participatory processes. It involved a panel of 51 experts and 30 stakeholders from different areas of knowledge and geographies. In three consecutive rounds the panel indicated their level of agreement or disagreement with indicator’s relevance for evaluating population health in Europe. Inferential statistics were applied to draw conclusions on observed level of agreement (Scott’s Pi interrater reliability coefficient) and opinion change (McNemar Chi-square test). Multivariate analysis of variance was conducted to check if the field of expertise influenced the panellist responses (Wilk’s Lambda test).

**Results:**

The panel participated extensively in the study (overall response rate: 80%). Eighty indicators reached group agreement for selection in the areas of: economic and social environment (12); demographic change (5); lifestyle and health behaviours (8); physical environment (6); built environment (12); healthcare services (11) and health outcomes (26). Higher convergence of group opinion towards agreement on the relevance of indicators was seen for lifestyle and health behaviours, healthcare services, and health outcomes. The panellists’ field of expertise influenced responses: statistically significant differences were found for economic and social environment (*p* < 0.05 in round 1 and 2), physical environment (*p* < 0.01 in round 1) and health outcomes (p < 0.01 in round 3).

**Conclusions:**

The high levels of participation observed in this study, by involving experts and stakeholders and ascertaining their views, underpinned the added value of using a transparent Web-Delphi process to promote agreement on what indicators are relevant to appraise population health.

**Electronic supplementary material:**

The online version of this article (10.1186/s12889-018-5463-0) contains supplementary material, which is available to authorized users.

## Background

Measuring population health is far from simple, beginning on which indicators to select and then how to aggregate them [1–6]. When choosing indicators to evaluate population health, it is important to consider a framework and a rationale for selection [7–11]. The understanding of what is population health may influence, at the first place, the type of indicators to be considered. Kindig and Stoddart ([12]: 320) argued that population health measurement should combine both the “health outcomes and their distribution within a population, the patterns of determinants that influence such outcomes, and the policies that influence the optimal balance of determinants”. The underlying assumptions behind the population health concept used by the authors have evolved from previous works in the 1990s, namely in Canada, putting the focus on the nonmedical determinants of health to understand why some populations are healthier than others and on their role to take action on population health improvement and reduction of health inequalities [13–15]. This perspective, described as a population health approach, is understood as an emerging discipline and as a concept extending public health model [16] by considering a wide range of health indicators and dimensions which are not part of traditional public health practice, such as education, income, and employment along with components of physical and built environments (e.g., urban design, housing, air quality) [12, 17].

The use of a set of core indicators from a wide range of health dimensions represents an asset for transparency and evidence-informed (health) policymaking [11, 18]. As Etches et al. [19] argue, health indicators can be used to advocate for different ideologies and are therefore not often employed neutrally. A major use is to measure the health of a population, to monitor inequalities and to determine whether or not expectations for performance are met, not only in the healthcare sector, but also in those other sectors which impact on health [20]. To accomplish this, indicators should be built on consensus [19]. Yet, the understanding of what constitutes a “good indicator” varies from scientist to politician to civil society [21, 22]. Different indicators can be selected, because different individuals will value and acknowledge certain aspects of health or determinants of health more than others [23].

Methods for selecting indicators could range from situations where researchers simply choose what they see as the most relevant indicators based on literature review [24] to participatory processes to help local communities identify their own indicators [25, 26]. Following this later path, a key issue to take into account in the indicator selection is assuring the involvement of a range of stakeholders [4, 23, 26, 27], who represent a variety of interests and knowledge, in addition to that of experts, as they have a good sense of the data issues and the scientific rigour regarding the proposed indicators [6, 28]. The use of participatory techniques increases the chance that the indicators selected will be deemed more credible, scientific and policy relevant, commonly understood and technically useful, which is directly linked with the need for health indicators reflecting substantial health problems and to be useful in guiding policy action [29].

Originally developed in the early 1950s as a way to assess expert opinion [30], the Delphi method is a well-known qualitative and structured technique to reach group agreement on a topic in systematic manner: as a result, it is used extensively in many different fields, including in health and social sciences [31–33]. According to Linstone and Turoff [34] the Delphi technique “may be characterized as a method for structuring a group communication process so that the process is effective in allowing a group of individuals, as a whole, to deal with a complex problem*”*. Thus, it has been commonly applied in indicator’s selection processes where group opinion is needed from an audience with varied views, such as in the health field. It has been used to select indicators which measure and monitor: i) quality and performance of healthcare services [35–40], ii) perinatal health in Europe [41] and iii) population health at national and municipal levels [6]. Its application on the identification of research priorities to address health inequalities [42] was recently reported as well. Because of its ease of application, the Delphi technique presents several advantages including: a) gathering opinions and knowledge of a wide range of individuals with diverse backgrounds and located in various regions and, b) ensuring anonymity [31, 34, 43].

The present study was undertaken as part of the EURO-HEALTHY, EU research project, which aims to advance knowledge on policies with the highest potential to promote health and health equity across European regions (http://www.euro-healthy.eu/). Underlying this project is the ground-breaking concept related to the multi- and trans-disciplinary approach and methods used to appraise population health and inequalities [44]. A multidimensional measure – the EURO-HEALTHY Population Health Index (PHI) – was designed to appraise population health in a wide range of health domains [45]. Under this approach, building the PHI combined: i) technical elements of a multicriteria model built with the Measuring Attractiveness by a Categorical Based Evaluation Technique (MACBETH) [46, 47] which is grounded upon the principles of multi-criteria value measurement [5, 48, 49], with ii) social elements of participatory processes for involving and capturing experts and stakeholders views on what is relevant for appraising population health (with these accessing summarised state of the art evidence). The first stage of the PHI construction focused on structuring, namely on the definition of areas of concern and their key dimensions and on the selection of a set of indicators to be included in the index.

To assure the integration of indicators from multiple areas of concern and dimensions, it was deemed critical that the following be combined: scientific evidence concerning health outcomes and health determinants affecting health, and the points of view of experts and stakeholders, with different backgrounds and expertise (including both the social and health sciences) and from diverse geographic contexts in Europe. Prior to the application of the Delphi method, a preliminary process was conducted to generate a list of potential indicators. This stage consisted of 1) an online survey to collect opinions from EURO-HEALTHY consortium researchers in the areas of concern and key dimensions viewed as relevant for analysing population health [50], and 2) a detailed literature review to identify indicators affecting health in each key dimension, made by researchers of thematic work packages [51–53].

Therefore, research reported in this article succeeds a phase of defining, based upon literature, a set of indicators of population health and is centred on the participatory process developed to involve a multidisciplinary panel of experts and stakeholders in the process of selecting indicators to evaluate population health at the European regional level. Attention is placed on the specific design of the Delphi process, on the analysis of results with inferential statistics, and on the implications of results for population health measurement research. In a later path, this set would be the basis for the construction of the EURO-HEALTHY Population Health Index.

## Methods

### Study design

A web-based Delphi process was developed to involve the experts and stakeholders and ascertain their views on the indicators considered relevant to appraise population health, with specific rules in place for dealing with differences in opinion and how to measure level of agreement. According to Delphi method literature, Delphi processes focus on opinion building (contributing towards consensus on a complex issue) and are characterised by: i) controlled feedback, where feedback of group opinion is provided after each round, ii) iteration of rounds, where each participant has the opportunity to revise his/her answers in light of the group opinion, and iii) anonymity. After reviewing the group statistics, each participant can decide whether to change or maintain his/her previous answer. The anonymity avoids unwillingness to abandon publicly expressed opinions and allows respondents not to fear group pressure when changing a previously expressed opinion [34, 43]. These features, as well as the possibility of engaging participants from distinct geographic locations, made the choice of a Delphi process appropriate for our context.

**Fig. 1:**
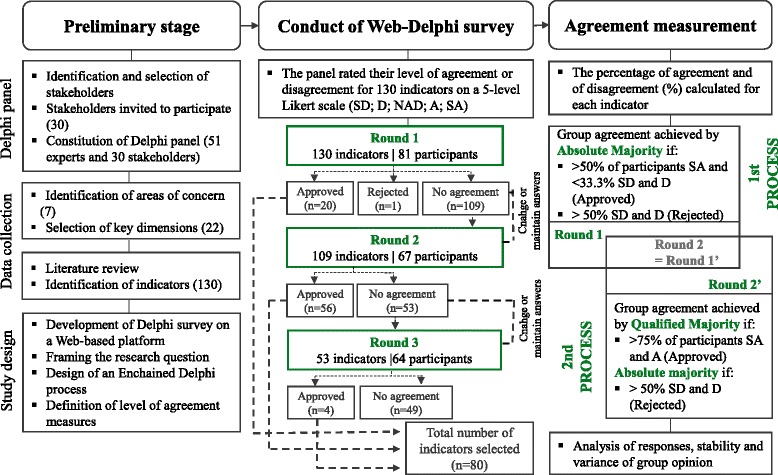
Flowchart of the Delphi process for selecting indicators. SD: Strongly disagree; D: Disagree; NAD: Neither agree nor disagree; A: Agree; SA: Strongly agree

Figure 1 shows the flow of information throughout the study, including the preliminary stage of generating the list of potential indicators, panel formation and study design, the carrying out of the Delphi survey, and the protocol implementation to measure the level of group agreement.

### Delphi panel

The panel for the purpose of this study was defined as a group of panellists with applicable knowledge in a variety of health domains and a keen level of interest in the field of European population health.

Since in Delphi studies, there are no general or specific rule for an optimal panel size, being very variable [34, 54, 55], the decision on the number of panellists was pragmatic, taking into consideration the aim of the study and resources available. An aspect considered relevant to be assured was the inclusion of all relevant perspectives, assessed by the qualities of the panel, rather than its numbers [56]. The involvement of both experts and stakeholders, with heterogeneous expertise and from diverse geographical regions, was deemed as criteria for constituting the panel in order to obtain a wider breadth of input from different scopes, from research to policy action.

- ■ Experts with knowledge on multiple dimensions of population health
  Experts were defined as researchers from diverse and wide-ranging areas who have substantial knowledge and information, from diverse and wide-ranging areas, on the multiple factors influencing population health and health inequalities at the European level. All of the 51 researchers (from 15 institutions of 12 European Union Member States), participating in the EURO-HEALTHY consortium were eligible and agreed to participate.
- ■ Stakeholders with the ability to influence policy
  A stakeholder mapping exercise was carried out at the start of the research, with the objective of identifying individuals that might have significant level of power and interest in population health, or considerable knowledge thereof [50]. The identification of potential stakeholders was based on different attributes, namely: i) their ability to influence policy at various decision levels (national, regional and metropolitan), ii) their scope for intervention (public sector, private sector and civil society), iii) their area of work (e.g. environment, public health, urban planning, groups at risk) and iv) geographic location (to reflect Europe’s diversity). Each of the 15 EURO-HEALTHY partners identified at least two stakeholders from the respective country, mainly working in the fields of health, urban planning and environment. Attention was given to stakeholders with comprehensive knowledge of at any at-risk population (e.g. migrants, Roma population) and representing national and regional/metropolitan level of decision. Before sending a formal invitation, each partner asked stakeholders their willingness to participate in the study as part of the Delphi panel. A total of 30 stakeholders were invited and agreed to participate.
  The Delphi panel was constituted by a total of 81 experts and stakeholders, reflecting a wide range of expertise profiles: Economics and health systems (9 panellists); Environmental health, ecological systems and sustainability (15 panellists); Epidemiology, social medicine and public health (29 panellists) and Health geography, demography and sociology (28 panellists). Furthermore, the panel reflects the Europe’s diversity, with a representation of the different geographical, political and socioeconomic contexts characterizing the regions of: Northern Europe (16 panellists from Sweden and United Kingdom), Western Europe (18 from Belgium, France, Germany and The Netherlands), Eastern Europe (12 from Czechia, Poland and Slovakia) and Southern Europe (35 from Greece, Italy, Portugal and Spain) [57].

### Web-Delphi survey

The literature collection process carried out previous to the Delphi – described in section 1 – yielded the identification of 130 potential indicators of health determinants and health outcomes, grouped within 7 areas of concern and 22 dimensions, which were then used to inform the Delphi process. These are listed in Additional file 1. The list of 130 indicators comprised 23 economic and social determinants (17.7%), 11 indicators of demographic change (8.5%), 10 representing lifestyle and health behaviours (7.7%), 22 indicators related to physical environment conditions (16.9%), 20 from built environment (15.4%), 12 indicators of healthcare services (9.2%) and 32 health outcomes (24.6%), encompassing mortality and morbidity indicators. The role of the panel was to review these indicators and state the level of agreement with regard to how relevant each indicator would be in evaluating Europe’s population health. Panellists were advised not to withhold their views about the potential relevance of an indicator on the basis of any perceived difficulties in collecting or processing the data required to calculate it. Therefore, each potential indicator was listed along with a short description entailing information about its calculation, data availability and comparability (across EU regions and over time) (Additional file 2).

**Fig. 2:**
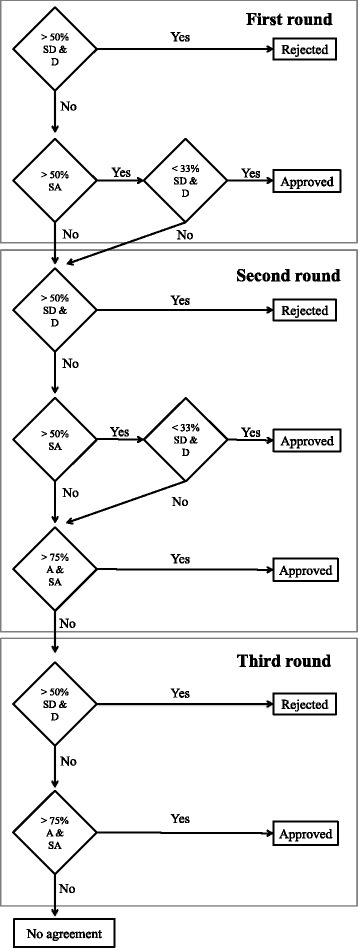
Flowchart of the decision rules adopted for indicator approval and rejection. SD: Strongly disagree; D: Disagree; NAD: Neither agree nor disagree; A: Agree; SA: Strongly agree

The Delphi survey was employed and conducted via a web platform (http://www.banaconsulting.com/delphiapp/Pages/LoginPage.aspx), which was established for implementing and monitoring the various EURO-HEALTHY web-based participatory processes. The need to adopt a flexible process in which there is an adjustment of the group agreement rules for selecting indicators along the Web Delphi led to the design of an innovative “enchained Delphi process”, which comprised two sub-processes, each one with two rounds in a total of three rounds: the second round of the 1st process was simultaneously the first round of the 2nd process (Figs. 1 and 2) [50, 58]. The Delphi was carried out between June and July 2015, starting with the panellist’s online invitation, containing individual credentials to log in to the web platform, information regarding the study design and the first round. The web platform was used to deliver the survey and to do follow-up of the process, providing feedback, in all rounds of the study (Additional file 2). Panellists were asked to answer within 2 weeks of receiving the e-mail. Up to two reminders were sent to those participants who did not complete the survey in the previously specified 2-week time frame. The panel members who did not respond to a round, within this period and after extended deadlines, were not invited to take part in further rounds.

The web-survey incorporated the list of potential 130 indicators, grouped by area of concern, along with information regarding the respective dimension, scientific relevance and data comparability (across EU regions and over time) [50].

In this first round and for each indicator, panellists were required to indicate their agreement or disagreement with the following statement “*This indicator is relevant to the evaluation of the Europe’s population health*”, on a 5-level Likert scale [59], with *Strongly agree* (SA) and *Agree* (A) indicating agreement, and *Strongly disagree* (SD) and *Disagree* (D) indicating disagreement. Additionally, panellists could indicate that they *Neither agree nor disagree* (NAD). A space in which participants could insert free-text comments was also available. In the second round each panel member was presented with the results of the first round, regarding those indicators approved and rejected that had achieved group agreement together with an individually tailored report with the panellist’s answer and the anonymous aggregation of all individual answers for each indicator. This enabled each panellist to consider his or her own answer with respect to the group opinion. The indicators that did not reach agreement were included for re-evaluation, with panellists taking the option to change or maintain their original answers for these indicators in light of the information provided, and with a view to contributing to greater agreement. In the third round the procedures applied in the previous round were replicated.

The group opinion (aggregate of individual opinions) was defined by calculating the percentage of responses, given in each Likert item, for each indicator in each round. The use of percentages is particularly meaningful in Likert scales and its definition can be based on standards such as political voting systems (e.g. simple majority, absolute majority) [43]. Group agreement, which could be meant to determine either for approval or rejection of any given indicator, was established by applying specific rules for dealing with differences in opinion, described below. The conceptualization of an enchained process enabled the establishment of decision rules for the 1st Delphi sub-process and the addition of new decision rules in the 2nd Delphi sub-process (Fig. 2). The number of majority agreements and disagreements was calculated by expressing the participants’ answers “SA”, “A” and “SD” and “D” in percentages per indicator. Therefore, during the 1st Delphi sub-process, those indicators receiving more than 50% of “SA” responses in the first round and which, at the same time, did not have more than 1/3 (< 33.3%) of “SD” and “D” responses, were immediately approved by *absolute majority*. Conversely, indicators with more than 50% of either “SD” or “D” responses were immediately rejected by *absolute majority*. In the 2nd Delphi sub-process, the rules for approval and rejection by *absolute majority*, as applied in the 1st Delphi sub-process, were kept. Yet, an additional rule, less strict, was put in place to allow for agreement on the selection of a high number of indicators: an indicator receiving more than 75% of “SA” and “A” responses was then approved by *qualified majority*. For the third round (corresponding to the second iteration of 2nd Delphi sub-process), group agreement for indicator approval was determined by *qualified majority*, maintaining the same rule for rejection (by *absolute majority*).

### Statistical analysis

Several statistical techniques, usually employed in Delphi studies [43, 60] were applied to the responses given by the panel across rounds, through which it was possible to analyse and describe the level of group agreement obtained for each indicator. Descriptive statistics were calculated: i) central tendency (mean and median) and ii) dispersion (coefficient of variation and interquartile range) measures. Inferential statistics were applied to draw conclusions on the Inter-Rater Reliability (IRR), stability and opinion change, and group’s opinion variance.

The IRR was computed for each indicator in each round using the Scott’s [61] Pi statistic [62], which is a kappa-like coefficient suitable for nominal data with three or more coders [63]. This coefficient measures the observed level of agreement between panellists and corrects for agreement that would be expected by chance, adding consistency to the analysis of agreement percentages by preventing overestimation of the level of agreement. This statistic ranges from − 1 to 1, with 1 indicating perfect agreement (consensus), 0 indicating completely random agreement, and − 1 indicating perfect disagreement (consensus). Values from 0.0 to 0.2 indicate slight agreement, 0.21 to 0.40 indicate fair agreement, 0.41 to 0.60 indicate moderate agreement, 0.61 to 0.80 indicate substantial agreement, and 0.81 to 1.0 indicating almost perfect or perfect agreement [63].

The stability of experts’ opinions between the successive rounds was verified through the application of the McNemar Chi-square test, which is a nonparametric statistic commonly used to quantify the degree of shift in responses, either in a positive (agreement) or in a negative (disagreement) direction, and to assess statistically significant changes (*P* values less than 0.05) [43]. Furthermore, an analysis was made of the contribution of opinion changes for the approval of indicators during the 2nd and 3rd rounds.

Finally, multivariate analyses of variance (MANOVA) were performed to test the group’s opinion variance and, in turn, to examine whether the responses given by the panel were statistically different across the following groups: i) type of panellist (expert vs. stakeholder) and ii) field of expertise (4 groups). Specifically, the Wilk’ Lambda test and associated F value were used to test the hypothesis that e.g. the field of expertise of a panellist (independent variable) has an effect on the response given in each indicator (dependent variable). A significance level of 95% (P values less than 0.05) was used to identify any pattern that is statistically significant. The post-hoc Tukey test was used for pairwise comparison of the mean response of the 4 groups of expertise in order to identify which ones differed significantly from the others.

## Results

### Panel participation

Regarding the panel participation, a high response rate was achieved from the experts and stakeholders: i) 88.9% in the 1st round (72 out of 81 participants); ii) 93.1% in the 2nd round (67 out of 72 participants) and iii) 95.5% in the last round (64 out of 67 participants) (Table 1). The drop-out rate was substantially higher for the panel of stakeholders (23.3% in the 1st round; 13% in 2nd round and 15% in the 3rd round) in comparison with the group of experts (3.9% in the 1st round; 4.1% in the 2nd round and 0% on the 3rd round). Considering the sub-groups representing different fields of expertise and geographical regions, the overall participation stands above 75%. Exception made for the panellists from environmental health, ecological systems and sustainability and from Northern Europe where, respectively, 60% and 68% of the invited individuals participated in all rounds.

**Table 1:** Participant response rate, by panellist characteristic

Characteristic	No.	No. (% drop-out rate)		
	Invited	Round 1	Round 2	Round 3
Total	81	72 (11.1)	67 (6.9)	64 (4.5)
Gender				
Male	37	33 (10.8)	31 (6.1)	30 (3.2)
Female	44	39 (11.4)	36 (7.7)	34 (5.6)
Type of panellist				
Expert	51	49 (3.9)	47 (4.1)	47 (0)
Stakeholder	30	23 (23.3)	20 (13.0)	17 (15.0)
Field of expertise				
Economics and health systems	9	9 (0)	8 (11.1)	8 (0)
Environmental health, ecological systems, sustainability	15	11 (26.7)	10 (9.1)	9 (10.0)
Epidemiology, social medicine and public health	29	26 (10.3)	23 (11.5)	22 (4.3)
Health geography, demography and sociology	28	26 (7.1)	26 (0)	25 (3.8)
Region of Europe				
Northern Europe	16	13 (18.8)	12 (7.7)	11 (8.3)
Western Europe	18	15 (16.7)	15 (0)	14 (6.7)
Eastern Europe	12	11 (8.3)	10 (9.1)	10 (0)
Southern Europe	35	33 (5.7)	30 (9.1)	29 (3.3)

### Indicators

From the list of proposed 130 indicators, a total of 80 indicators (61.5%) reached group agreement for selection and were endorsed as relevant to appraise population health in the following areas of concern: Economic and social environment – ESE (12); Demographic change – DC (5); Lifestyle and health behaviours – LHB (8); Physical environment – PHE (6); Built environment – BTE (12); Healthcare services – HCS (11) and Health outcomes – HO (26). One indicator achieved agreement for rejection and about one third of indicators presented lack of agreement. The large majority of the indicators proposed in the areas of concern of LHB, HCS and HO were approved by 80%, 91.7% and 81.3%, respectively. As for the PHE indicators, 72.7% of the PHE indicators presented lack of agreement, either for approval or rejection (Table 2). In summary, 36 indicators were approved by *absolute majority* (SA > 50% and SD + D < 33.3%), 44 by *qualified majority* (SA + A > 75%) and only 1 indicator was rejected by *absolute majority* (SD + D > 50%) (Table 3).

**Table 2:** Number of indicators proposed, approved and rejected, by area of concern and key dimension

Area of concern and dimension	Proposed	Group agreement		No agreement
		Approved	Rejected	
Economic and social environment (ESE)	23	12	1	10
Employment	4	3	0	1
Income & living conditions	7	4	0	3
Social protection	4	1	0	3
Education	3	3	0	0
ICT access & use	1	0	0	1
Governance	3	0	1	2
Security	1	1	0	0
Demographic change	11	5	0	6
Migration	5	2	0	3
Ageing	3	3	0	0
Population change	3	0	0	3
Lifestyle and health behaviours	10	8	0	2
Physical environment	22	6	0	16
Pollution	16	6	0	10
Extreme weather events	6	0	0	6
Built environment	20	12	0	8
Housing conditions	6	5	0	1
Land use and transport	2	0	0	2
Water and sanitation	5	4	0	1
Waste management	3	0	0	3
Road safety	4	3	0	1
Healthcare services	12	11	0	1
Healthcare resources	6	6	0	0
Healthcare access	2	1	0	1
Healthcare utilization	1	1	0	0
Healthcare expenditure	3	3	0	0
Health outcomes	32	26	0	6
Length of life (Mortality)	25	20	0	5
Quality of life (Morbidity)	7	6	0	1
Total	130	80	1	49

**Table 3:** Number of indicators approved and rejected, by group majority decision rules

Area of concern	A1-A	A2-A	A2-B	A3-B	R1-A
Economic and social environment	4	0	8	0	1
Demographic change	0	0	5	0	0
Lifestyle and health behaviours	4	2	2	0	0
Physical environment	0	2	3	1	0
Built environment	0	5	6	1	0
Healthcare services	0	1	9	1	0
Health outcomes	12	6	7	1	0
Total	20	16	40	4	1

*A1-A* Approved in Round 1, by Absolute Majority (SA = > 50% and SD + D < =33.3%), *A2-A* Approved in Round 2, by Absolute Majority (SA= > 50% and SD + D < =33.3%), *A2-B* Approved in Round 2, by Qualified Majority (SA + A = > 75%), *A3-B* Approved in Round 3, by Qualified Majority (SA + A = > 75%), *R1-A* Rejected in Round 1, by Absolute Majority (SD + D = > 50%)

The addition of the decision rule of *Qualified Majority* in the 2nd Delphi sub-process resulted in a higher number of approved indicators in all areas of concern (55% of indicators were approved by this rule), namely in the areas of concern of ESE and HCS. Additional file 3 presents the Delphi results for each indicator proposed, with the aggregated responses from the panel, summarized by central tendency and dispersion statistics (mean, median, coefficient of variation and interquartile range) and observed level of agreement (Scott’s Pi statistic).

#### Level of agreement by area of concern

**Fig. 3:**
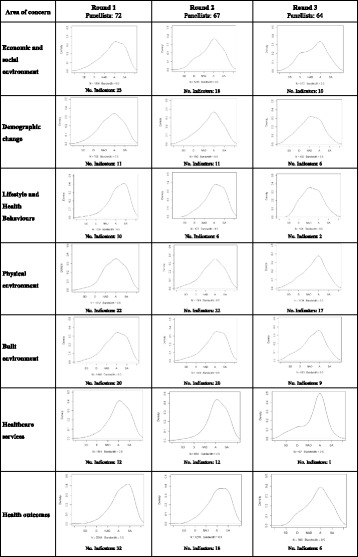
Panellist’s vote distribution curve over successive Delphi rounds, by area of concern (Kernel density curves of responses). Each density curve represents the distribution of the panellist’s responses on a 5-level Likert scale (SD: Strongly disagree; D: Disagree; NAD: Neither agree nor disagree; A: Agree; SA: Strongly agree) by area of concern and round. In each round the density curve of responses given on the indicators included for evaluation is presented. In the 2nd and 3rd rounds, the vote distribution curve corresponds to the answers given on the indicators that did not gather agreement in the 1st round and were included for re-evaluation

**Fig. 4:**
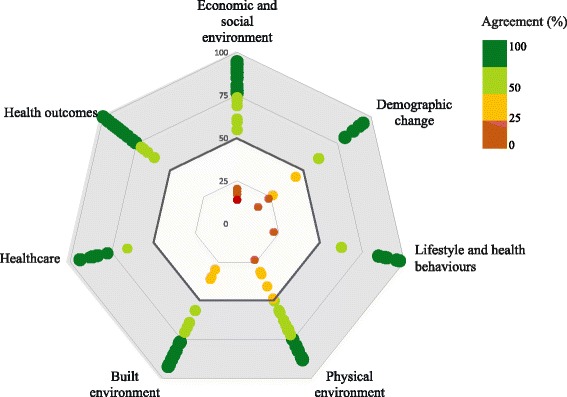
Radar chart of level of agreement (%) achieved by each indicator, by area of concern, after the 3rd round. The data plot includes the final percentage agreement (statistical aggregation of Agree and Strongly Agree responses) achieved by each indicator, at the end of the Delphi process. Percentage agreement for each indicator is shown as individual coloured dots. The grey area represents indicators where more than 50% of the panellists agree or strongly agree with its relevance. It adds information to the assessment of the overall agreement achieved, not reflecting the application of the decision rules

Figure 3 shows the kernel density curves of responses in each area of concern over the three rounds. Denser distributions towards the right of the scale indicate agreement and toward the left indicate disagreement regarding the inclusion of the indicators. Leptokurtic (tall and thin) distributions represent higher agreement among the members of the panel whereas platykurtic (flatter) distributions indicate lower agreement. During the 1st and 2nd rounds, all curves present one distinct high point or peak, at the right of the scale, indicating that the majority of respondents tend to agree and strongly agree with the relevance of the respective indicators. In the 3rd round, the distribution of responses on the areas of concern of ESE, DC and LHB indicate less agreement and none of the indicators proposed gathered the agreement needed for approval. After the 3 rounds of the enchained Delphi process, the level of agreement achieved by each indicator (statistical aggregation of Agree and Strongly Agree responses) revealed a higher convergence of group opinion towards higher agreement on the relevance of indicators from the LEB, HO and HCS areas of concern (Fig. 4).

Table 4 presents the percentage of agreement reached on the indicators approved by group majority, by area of concern and key dimension. In total, 80 indicators achieved group agreement regarding their relevance to appraise population health in 18 dimensions, covering 7 areas of concern for population health. The proposed indicators within the dimensions of “ICT access and use”, “Governance”, “Population change”, “Extreme weather events” and “Waste management” did not reach agreement, and were therefore removed from the final list.

**Table 4:** Indicators approved and level of agreement achieved, in each area of concern and dimension

Dimension	Indicator	Aggregated responses (%)					*Pi*	Group majority decision rule
		SD	D	NAD	A	SA		
Economic and social environment								
Employment	Unemployment rate (%)	1.4	1.4	2.8	30.6	63.9	0.50	A1-A
	Youth unemployment rate (%)	0.0	3.0	9.0	49.3	38.8	0.39	A2-B
	Long-term unemployment rate (%)	1.4	4.2	4.2	25.0	65.3	0.49	A1-A
Income & living conditions	Disposable income of private households per capita (€)	0.0	3.0	11.9	50.7	34.3	0.38	A2-B
	People at risk of poverty or social exclusion (%)	1.4	1.4	4.2	20.8	72.2	0.56	A1-A
	Severe material deprivation rate (%)	2.8	2.8	4.2	38.9	51.4	0.41	A1-A
	Disposable income ratio - S80/S20	1.5	4.5	16.4	34.3	43.3	0.32	A2-B
Social protection	Expenditure on care for elderly (% of GDP)	0.0	9.0	14.9	47.8	28.4	0.33	A2-B
Education	Population aged 25–64 with lower secondary education attainment (%)	0.0	14.9	7.5	46.3	31.3	0.33	A2-B
	Population aged 25–64 with upper secondary or tertiary education attainment (%)	0.0	9.0	10.4	52.2	28.4	0.36	A2-B
	Early leavers from education and training (%)	0.0	9.0	13.4	41.8	35.8	0.32	A2-B
Security	Population who reported crime, violence or vandalism in the area of residence (%)	0.0	6.0	7.5	50.7	35.8	0.39	A2-B
Demographic change								
Migration	Immigrants at risk of poverty or social exclusion - born in EU28 countries, excluding the reporting country (%)	1.49	4.5	7.5	61.2	25.4	0.44	A2-B
	Immigrants at risk of poverty or social exclusion - born in non EU28 countries (%)	0	1.5	7.5	58.2	32.8	0.44	A2-B
Ageing	At risk of poverty rate of older people - aged 65 years or over (%)	0	1.5	4.5	50.7	43.3	0.44	A2-B
	Ageing index	0	6	7.5	40.3	46.3	0.38	A2-B
	Age dependency ratio	0	7.5	12	50.7	29.9	0.36	A2-B
Lifestyle & health behaviours								
Lifestyle and health behaviours	Adults who are obese (%)	0.0	1.4	1.4	25.0	72.2	0.58	A1-A
	Regular daily smokers in the population - aged 15 and over (%)	2.8	4.2	4.2	22.2	66.7	0.49	A1-A
	Daily smokers - aged 15 and over (%)	2.8	6.9	5.6	27.8	56.9	0.40	A1-A
	Number of cigarettes smoked per day - daily smokers (No.)	0	6	4.5	38.8	50.7	0.40	A2-A
	Pure alcohol consumption - aged 15 and over (Liters per capita)	0.0	0.0	4.2	43.1	52.8	0.46	A1-A
	Population engaged in vigorous or moderate physical activity on 2 or more days a week (%)	0	0	4.5	50.7	44.8	0.45	A2-B
	Average amount of fruits and vegetables available per person per year (Kg per person)	0	0	12	55.2	32.8	0.42	A2-B
	Live births by mothers under age of 20 (%)	0	4.5	7.5	34.3	53.7	0.41	A2-A
Physical environment								
Pollution	Annual mean of the daily PM2.5 concentrations (ug/m3)	0	0	13	34.3	52.2	0.40	A2-A
	Annual mean of the daily PM10 concentrations (ug/m3)	0	0	12	37.3	50.7	0.40	A2-A
	Transport emissions of air pollutants (%)	7.46	4.5	4.5	58.2	25.4	0.40	A2-B
	Population exposed to traffic noise - Lden 55-59db, during day (%)	6.25	3.1	11	73.4	6.25	0.55	A3-B
	Population who reported pollution, grime or other environmental problems in the area of residence (%)	2.99	1.5	16	43.3	35.8	0.33	A2-B
	Contaminated sites and other land use indicators (hectares)	1.49	1.5	15	47.8	34.3	0.36	A2-B
Built environment								
Housing conditions	Average number of rooms per person	1.56	6.3	16	53.1	23.4	0.36	A3-B
	Children living in homes with problems of dampness (%)	0	0	9	53.7	37.3	0.43	A2-B
	Population living in a dwelling with a leaking roof, damp walls, floors or foundation, or rot in window frames of floor (%)	0	3	12	28.4	56.7	0.41	A2-A
	Households without indoor flushing toilet (%)	0	3	9	31.3	56.7	0.42	A2-A
	Households without central heating (%)	0	0	15	23.9	61.2	0.45	A2-A
Water and sanitation	Population connected to public water supply (%)	0	4.5	4.5	49.3	41.8	0.41	A2-B
	Drinking water quality: microbiological non-compliance (%)	0	4.5	3	35.8	56.7	0.44	A2-A
	Drinking water quality: chemical non-compliance (%)	7.46	4.5	4.5	37.3	46.3	0.35	A2-B
	Population connected to wastewater treatment (%)	0	4.5	3	40.3	52.2	0.43	A2-A
Road safety	Injury rate due to road traffic accidents, per 100.000 inhabitants	0	4.5	7.5	40.3	47.8	0.39	A2-B
	Victims in road accidents - injured and killed, per 100.000 inhabitants	0	7.5	15	32.8	44.8	0.33	A2-B
	Fatality rate due to road traffic accidents, per 1.000 victims	0	6	12	37.3	44.8	0.35	A2-B
Healthcare services								
Healthcare resources	Health personnel (general practitioners), per 100.000 inhabitants	1.49	1.5	3	52.2	41.8	0.44	A2-B
	Curative care beds, per 100.000 inhabitants	0	9.4	13	57.8	20.3	0.39	A3-B
	Long-term care beds in nursing and residential care facilities, per 100.000 inhabitants	1.49	3	18	47.8	29.9	0.34	A2-B
	Medical doctors, per 100.000 inhabitants	1.49	3	12	32.8	50.7	0.37	A2-A
	Health personnel (nurses and midwives, dentists, pharmacists and physiotherapists), per 100.000 inhabitants	1.49	3	12	35.8	47.8	0.36	A2-B
	Medical Technology (e.g. mammography, PET scanners, CT scanners) per 100.000 inhabitants	1.49	4.5	12	50.7	31.3	0.36	A2-B
Healthcare access	Population living to more than 60 min from Urgent Care/Emergency unit (%)	0	6	9	40.3	44.8	0.36	A2-B
Healthcare utilization	Hospital discharges due to diabetes, hypertension and asthma per 100.000 inhabitants	0	3	10	55.2	31.3	0.41	A2-B
Healthcare expenditure	Health care expenditure by all financing agents per inhabitant, in Purchasing Power Standards (PPS)	2.99	1.5	10	56.7	28.4	0.41	A2-B
	Health care expenditure by private household out-of-pocket expenditure per inhabitant, in PPS	0	0	12	55.2	32.8	0.42	A2-B
	Governmental (except social security funds) expenditure in providers of health care in health care per capita, in PPS	1.49	0	12	46.3	40.3	0.38	A2-B
Health outcomes								
Length of life (Mortality)	Life expectancy at birth (years)	0.0	0.0	0.0	25.4	74.6	0.62	A1-A
	Sex ratio of life expectancy at birth	0	9.4	13	57.8	20.3	0.39	A3-B
	Life expectancy at 65 years of age (years)	0.0	0.0	4.2	31.0	64.8	0.51	A1-A
	Infant mortality, per 1.000 live births	0.0	2.8	4.2	18.3	74.6	0.59	A1-A
	Perinatal mortality, per 1.000 live births	0	3	11	28.8	57.6	0.42	A2-A
	Neonatal mortality, per 1.000 live births	0	3	12	28.8	56.1	0.40	A2-A
	Premature mortality, SDR per 100.000 inhabitants	0.0	1.4	9.9	23.9	64.8	0.48	A1-A
	Amenable deaths to health, SDR per 100.000 inhabitants	0	1.5	14	42.4	42.4	0.37	A2-B
	Preventable deaths, SDR per 100.000 inhabitants	0.0	1.4	5.6	42.3	50.7	0.43	A1-A
	Deaths related to infectious disease, SDR per 100.000 inhabitants	0	1.5	4.5	40.9	53	0.44	A2-A
	Deaths related to land transport accidents, SDR per 100.000 inhabitants	1.52	6.1	11	40.9	40.9	0.34	A2-B
	Deaths from diseases of the circulatory system, SDR per 100.000 inhabitants	0.0	0.0	8.5	33.8	57.7	0.45	A1-A
	Deaths from diseases of the respiratory system, SDR per 100.000 inhabitants	0.0	0.0	5.6	38.0	56.3	0.46	A1-A
	Deaths from cancer (malignant neoplasms), SDR per 100.000 inhabitants	0	0	3	42.4	54.5	0.47	A2-A
	Death due to ischaemic heart disease, SDR per 100.000 inhabitants	0.0	0.0	4.2	39.4	56.3	0.47	A1-A
	Deaths due to cerebrovascular diseases, SDR per 100.000 inhabitants	0.0	0.0	7.0	36.6	56.3	0.45	A1-A
	Deaths due to colorectal cancer, SDR per 100.000 inhabitants	0.0	0.0	7.0	42.3	50.7	0.43	A1-A
	Deaths due to larynx, trachea, bronchus and lung cancer, SDR per 100.000 inhabitants	0.0	0.0	8.5	40.8	50.7	0.42	A1-A
	Deaths due to breast cancer, SDR per 100.000 inhabitants	0.0	2.8	7.0	39.4	50.7	0.41	A1-A
	Deaths due to prostate cancer, SDR per 100.000 inhabitants	1.52	6.1	4.5	39.4	48.5	0.39	A2-B
Quality of life (Morbidity)	People who reported having a long-standing illness or health problem (%)	0	3	6.1	37.9	53	0.42	A2-A
	Self-perceived health less than good (%)	0	7.6	14	31.8	47	0.34	A2-B
	Self-reported unmet needs for medical examination (%)	1.52	6.1	17	47	28.8	0.32	A2-B
	Age-standardized Disability-Adjusted Life Year (DALY) rate	1.52	1.5	9.1	24.2	63.6	0.46	A2-A
	Low birth-weight (%)	0	6.1	12	42.4	39.4	0.34	A2-B
	Preterm birth (%)	0	6.1	15	37.9	40.9	0.33	A2-B

*SD* Strongly disagree, *D* Disagree, *NAD* Neither agree nor disagree, *A* Agree, *SA* Strongly agree, *Pi* Scott’s Pi inter-rater reliability coefficient, *A1-A* Approved in Round 1 - 1st Delphi process, by Absolute Majority (SA > 50% and SD + D < 33.3%), *A2-A* Approved in Round 2 - 1st Delphi process, by Absolute Majority (SA > 50% and SD + D < 33.3%), *A2-B* Approved in Round 2 - 2nd Delphi process, by Qualified Majority (SA + A > 75% and SD + D < 33.3%), *A3-B* Approved in Round 3 - 2nd Delphi process, by Qualified Majority (SA + A > 75%)

The observed level of agreement between panellists (*Pi* - Scott’s Pi inter-rater reliability coefficient) indicates substantial and moderate agreement in 40 indicators (*Pi* between 0.41 and 0.62) and fair agreement in 41 indicators (*Pi* between 0.32 and 0.40). The majority of indicators presenting fair agreement were approved by qualified majority during the 2nd Delphi process.

### Stability and group opinion variance

The stability of the distribution of the group’s response was calculated for indicators that did not reach agreement in the first and second rounds. A comparison of individual responses between rounds showed responses from Round 1 to Round 2 to be stable (not significantly changed) in 107 indicators (98.2%) as measured by the McNemar Chi-square test. This means that the majority of respondents (77%) did not make a significant change in the indicators included for re-evaluation, either in a positive (from disagreement to agreement) or a negative (from agreement to disagreement) direction. The stability of the group opinion increased from Round 2 to Round 3, where all indicators presented more stable responses and no statistically significant changes. However, the Delphi results show that changes in the opinion contributed to higher agreement, as observed by an increase in agreement on the indicators approved on Rounds 2 and 3. This was particularly obvious for the indicators of “Immigrants at risk of poverty or social exclusion” and “Children living in homes with problems of dampness (%)”, approved by qualified majority in the second round.

The analysis of variance on the distribution of the group’s response along the three rounds, provided relevant insights as to how the characteristics of the Delphi panel had an effect on the responses given in each indicator. First we conducted a multivariate analysis of variance to test whether the two groups of panellists (experts and stakeholders) are statistically different from each other in terms of their perceptions about the relevance of each indicator to appraise population health. Our null hypothesis was H0^1^: Perceptions about the relevance of each indicator do not vary as a function of the type of panellist. The analysis of Wilk’s Lambda and associated F value revealed a non-significant main effect in all rounds. Since *p*-value > 0.05, we cannot reject the null hypothesis at 5% significance level and conclude that the responses do not vary as a function of the panellist type. Next, we conducted MANOVA to test whether the responses of the four groups of expertise (economics and health systems; environmental health, ecological systems and sustainability; epidemiology, social medicine and public health; and health geography, demography and sociology) are statistically different from each other. The analysis revealed a significant main effect of field of expertise in the second round. Since p-value < 0.05 we rejected the null hypothesis and concluded that the responses varied as a function of the panellist field of expertise. The MANOVA analysis showed significant differences of opinion within the ESE area of concern (Rounds 1 and 2), the PHE area of concern (Round 1) and the HO area of concern (Round 3) (Table 5).

**Table 5:** MANOVA results: effect of expertise field on the responses within each area of concern

Area of concern	Round 1			Round 2			Round 3		
	Wilks’ Lambda	F value	P-value	Wilks’ Lambda	F value	P-value	Wilks’ Lambda	F value	P-value
Economic and social environment	0.159	1.703	0.004^a^	0.235	1.596	0.016^a^	0.563	1.082	0.366
Demographic change	0.609	0.952	0.548	0.584	0.952	0.549	0.642	1.469	0.108
Lifestyle and health behaviours	0.640	0.953	0.542	0.584	0.952	0.549	0.927	0.764	0.600
Physical environment	0.140	1.994	0.000^a^	0.199	1.370	0.066	0.306	1.263	0.147
Built environment	0.364	0.989	0.508	0.400	0.791	0.846	0.690	0.765	0.790
Healthcare services	0.509	1.206	0.215	0.510	1.097	0.341	0.859	1.552	0.167
Health outcomes	0.116	1.191	0.188	0.210	1.481	0.033	0.453	2.312	0.002^a^

^a^Statistically significant at 5% significance level

Based on the Tukey test, it was possible to identify the indicators where the mean values of panellist responses varied in function of the expertise field and to determine the group of panellists that differed significantly from the others. Table 6 presents the results of the pairwise comparisons for the three rounds. In the first round, significant differences of responses between groups of expertise were identified for the indicators of Physical environment, with panellists from the EHS field displaying different response behaviour in the majority of indicators. The response given by this group varied significantly from the response given by the EHESS and HGDS groups. In the second round, statistically significant differences in the mean responses were found in the ESE area of concern. The panellists from the EHS group of expertise rated the indicators of “Gross Domestic Product” and “Early leavers from education and training” differently from the ESMPH and HGDS panellists. In the final round, observed response differences were found in four HO indicators, mainly between the EHS and HGDS and between the EHESS and HGDS groups.

**Table 6:** Comparison of responses between the four groups of expertise, in all rounds

Round	Area of concern	Indicator	Field of expertise			
			EHS	EHESS	ESMPH	HGDS
1	ESE	Population who reported crime, violence or vandalism in the area of residence	●		●	
	PHE	Annual mean concentrations of Particulate Matter - PM2.5 (ug/m3)	●|□	●		□
	PHE	Annual mean concentrations of Particulate Matter - PM10 (ug/m3)	●|□	●		□
	PHE	Annual mean ozone concentrations	●|□|▲|♦	□	▲	♦
	PHE	Annual mean Nitrogen Dioxide concentration	●|□	●	▲	□|▲
	PHE	Annual mean Sulphur Dioxide concentration	●|□	●	▲	□|▲
	PHE	Number of high temperature days	●|□	●|▲	▲|♦	□|♦
	PHE	Number of low temperature days	●	●		
	PHE	Number of warm nights	●|□	● |▲	▲|♦	□|♦
	PHE	Number of heatwaves	●|□	● |▲	▲|♦	□|♦
	PHE	Heating degree days	●	●|□	□|♦	♦
2	ESE	Gross Domestic Product, per capita in Purchasing Power Standards (PPS)	●		●	
	ESE	Early leavers from education and training (%)	●			●
3	HO	Sex ratio of life expectancy at birth	●	□		●|□
	HO	Sex ratio of premature mortality	●	□		●|□
	HO	Sex ratio of amenable mortality		●		●
	HO	Deaths related to dementias including Alzheimer’s disease, SDR per 100.000 inhabitants			●	●

*EHS* Economics and health systems, *EHESS* Environmental health, ecological systems and sustainability, *ESMPH* Epidemiology, social medicine and public health, *HGDS* Health geography, demography and sociology. The symbols identify the groups where responses were found to be statistically different. Pairs of equal symbols identify the groups where panelist’s response differ significantly from each other. Example: the responses given by the EHS group of panelists (●|□) on the indicator “Annual mean concentrations of Particulate Matter – PM2.5 (ug/m3)” differed significantly from the answers presented by the EHESS (●) and the HGDS (□) groups

## Discussion

The main implications of the findings of the study will be discussed taking into consideration the following sections: i) level of agreement achieved on the relevance of indicators by area of concern, ii) participatory approach and the use of Delphi technique, iii) stakeholders and experts opinion variance, iv) strengths and limitations and v) further research.

### Level of agreement achieved on the relevance of indicators by area of concern

The Delphi process was successful in promoting agreement on a comprehensive set of indicators of health determinants and health outcomes. In this regard, a multidisciplinary panel (51 experts and 30 stakeholders) was consulted in three (enchained) rounds and, from a list of 130 potential indicators, 80 were approved. On the other hand, 50 indicators were considered not relevant taking into account the group majority rules applied, that is, they were rejected or do not achieved the group agreement needed to be selected. The panel reached agreement on indicators from multiple areas of concern and dimensions, underpinning the holistic and multidimensional approach of population health measurement. Indicators of health outcomes, namely that of the life expectancy at birth, infant mortality, premature mortality, and amenable and preventable mortality, attained a high level of agreement amongst the panellists. The area of concern of health outcomes comprised the highest number of indicators selected, with 81% of proposed indicators having been approved by group majority. For instance, life expectancy at birth reached substantial observed agreement between panellists (Pi = 0.62). This indicator is often taken as an overall measure of health and is frequently used in monitoring the health of populations over time [64, 65].

Along with health outcomes, indicators of lifestyle and health behaviours and of healthcare services achieved higher convergence on group opinion about their relevance on population health measurement. Of the proposed indicators within the areas of concern mentioned above, about 80% and 92% were selected, respectively. Substantial observed agreement was reached on indicators related with unhealthy habits, namely obesity (adults who are obese) [66], smoking (daily smokers – aged 15 and over) [67] and alcohol consumption [68].Their effects are widely recognized as health risks and strongly associated with an increased burden of disability, as extensively reported on the literature [69].

Regarding economic and social determinants of health, core indicators of employment (e.g. unemployment rate), income (e.g. people at risk of poverty or social exclusion) and education (e.g. population having attained upper secondary or tertiary education attainment) achieved group agreement as to their relevance to evaluate population health, confirming what is widely stated in the literature [70–72]. Many of these are already routinely collected and compiled in European countries and are effective tools for monitoring living conditions and overall wellbeing. In contrast, 43.5% of the proposed indicators economic and social conditions did not gather enough agreement to be selected, as a result of not achieving agreement by neither absolute majority nor qualified majority. Within this group, the indicators of dimensions of ICT access and use (households with access to Internet) and of governance (voter turnout in elections and reported satisfaction with democracy) were not considered pertinent to appraise population health for European regions. Other dimensions where uncertainty about the relevance of the indicators was high were not included in the final set selected. Lack of agreement emerged in 73% and 40% of the indicators proposed within the physical and built environment, respectively. For instance, high differences in opinion arose for all indicators of extreme weather events (physical environment), land use and transport and waste management dimensions (built environment), which appears to be in contrast with several studies in the literature that report the relationship between environmental conditions and population health outcomes [73–76]. Specifically, significant differences in responses were identified for extreme weather events indicators, where panellists’ expertise potentially had an effect on the response behaviour. In fact, concerns about the specificity, usefulness and data availability of environmental indicators at regional level were reflected in comments provided by various panellists and partly explain those results. Additionally, potential problems related with redundancy and collinearity of some indicators (e.g. regular daily smokers and daily smokers) were also identified in the comments provided across the Delphi.

### Participatory approach and the use of the Delphi technique

As for the method implemented for the selection of indicators, the Delphi technique was chosen as opposed to a standard questionnaire because it enhanced decision-making in a systematic manner [31, 56] and because of its ease of application through the use of an online survey. Key components to a Delphi process, including anonymity, iteration, controlled acquisition of feedback, and analytic aggregation of responses, were followed. There was a high response rate from experts and stakeholders and round-to-round dropout rates decreased as the Delphi process progressed: 11.1% in the 1st round, 6.9% in the 2nd round and 4.5% in final last round. Yet, no specific guidelines exist for an acceptable response rate and a number of authors recommend a 70% rate as necessary for each round to maintain rigor [77]. In our study, response rates exceeded 88% in all rounds.

The management of responses and nonresponses is a critical aspect in all Delphi studies. The facilitator is responsible for the administration of the Delphi process’s playing a fundamental role in its success [78]. The use of a web platform, to deliver the survey and to follow-up on the process, increased the efficiency of the facilitator and the Delphi procedures, easing data entry, responses and analysis. It simplified the process of gathering information from the panel, and enhanced the controlled opinion feedback and communication across rounds. The number and the technical quality of the answers were constantly monitored. The iteration of rounds, being the most distinctive characteristics of the Delphi technique, requires the continuous participation of a stable group of participants to provide their opinions in sequential rounds, with each round presenting group feedback from the previous [43]. For this reason, the panellists who did not respond to the first round were not invited to take part of further rounds. This allowed the participants’ responses be continuously assessed and integrated into the group feedback from the first to the last round.

The “retention” and commitment of participants throughout the consecutive rounds are key for the success of a Delphi. Yet, these processes could be much time consuming and require much effort from the participants. The literature shows that there are many reasons for abandoning a Delphi process: lack of time; strong deviation between individual opinion and the group opinion; uncertainty or perception of incompetency to answer to the topic, etc. [55]. During the whole participatory process six reminders (two per round) were sent to participants who did not complete the survey in the previously specified time allotted and deadlines were extended in some cases. This measure reduced the occurrence of high drop-out rates and the effect of non-response bias, recognizing that if the systematic drop-out of a certain sub-group of participants occurred, it would compromise the quality of results and the representativeness of all points of view [77]. Overall, the non-participation of 17 individuals potentially did not affect the final outcome of the indicator selection process, namely the level of group agreement. As mentioned before, care was taken to assure the representativeness of the different sub-groups in all rounds of the survey (e.g. experts and stakeholders; from four fields of expertise and from four geographical regions).

In this study, the level of agreement was used rather than consensus concept because it is less strict and easily interpretable, being consensus therefore a special case of agreement (perfect agreement) [79]. Group agreement, which could be either for approval or rejection of an indicator, was quantified by percentage and defined as being of absolute majority and qualified majority. Respectively, a level of 50% and 75% agreement or disagreement was considered as clear evidence of a majority opinion. The use of percentage measures to measure the level of agreement among a panel is common in Delphi studies literature [43] although there is no unanimity on what percentage of participant responses constitutes an acceptable level of agreement [54].The conceptualization of an enchained Delphi with two sub-processes (the second round of the 1st sub-process was simultaneously the first round of the 2nd sub-process) rather than one single process enabled to set specific decision rules of group majority for the 1st Delphi sub-process (absolute majority) and add a new rule in the 2nd Delphi sub-process (qualified majority), maximizing the number of indicators approved and assuring the selection of indicators in all areas of concern. In total, more than a half of the indicators were approved by qualified majority; indicators from demographic change were all approved by this rule, in the second round.

Overall, 80 (61.5%) of 130 indicators reached agreement for approval and one for rejection by the end of Delphi process, with 49 (37.7%) remaining with lack of agreement. Three rounds proved sufficient to attain stability in the responses, as shown by the McNemar Chi-square test results between Rounds 2 and 3. A panellist’s contemplation of whether or not to change opinion between rounds is a prerequisite for promoting a high-quality group opinion [80]. A key to successful Delphi process is to enable some panellists to change their opinion as a result of considering the views of their peers. In each round, panellists had the opportunity to change or maintain their original answers in light of the analytic aggregation of responses and in view to contributing to achieve agreement. The revision of answers and resulting opinion changes led to an increase in agreement percentages regarding the indicators approved in Rounds 2 and 3. As Bolger et al. [81] found in his study, the majority opinion was the strongest influence on panellists’ change of opinion.

### Stakeholders and experts opinion variance

This study has investigated the views of scientific experts and stakeholders on the relevance of multiple indicators for the evaluation of European population health. The fact that the Delphi panel was comprised by different types of panellists - experts and stakeholders - and from heterogeneous fields of expertise and geographical regions enhanced further differentiation in the analysis and the quality of the results, leading us to verify if the differences in opinion varied as a function of these characteristics. As reported by Makkonen, Hujala & Uusivuori [80], Delphi respondents could include not only neutral experts but also experts with high stakes, predetermined opinions and motivations. Thus, the intent was to study the effect of panellists’ expertise on perceptions about relevant indicators to appraise population health. The multivariate analysis of variance revealed that the perceptions about the relevance of each indicator did not vary as a function of the type of panellist – experts and stakeholders. In contrast, the mean values of panellist responses, within each area of concern, varied with regard to background, thus making it possible to identify the group of panellists that showed differing response behaviour from the others. Significant differences of opinion were found for indicators within the areas of concern of Economic and social environment, Physical environment and Health outcomes. Panellists from the economics and health systems field of expertise presented a mean of responses significantly different from the other groups in the majority of indicators. In terms of results, these differences in perceptions reflects the importance of having a panel of experts and stakeholders with different backgrounds. The field of expertise of a panellist may play a key role in how he or she understands the relevance of a certain indicator for appraising population health. These results would then call decision-makers’ attention to the importance of engaging stakeholders from different fields of expertise in policy development on health as a factor to assure the quality and trustworthiness of those policies that are aimed at improving health.

### Strengths and limitations

To our knowledge this is the first study in which the Delphi method using a web platform was employed as part of the selection process to set up a comprehensive list of indicators relevant to appraise population health in multiple dimensions. Across the Web Delphi process, a high response rate was achieved over three rounds, with 88.9%, 93.1% and 95.5% obtained during the first, second and third rounds, respectively. These values represent very high peaks of response rates for Delphi studies and thus should be considered one of the strengths of the process since it implied low non-response bias, as documented in other studies [40]. Almost 80% of the invited panellists participated in all rounds and completed the Delphi survey, exceeding expectations considering it was a large and diverse panel, characteristics reported in the literature as factors of higher attrition rates and withdrawal during the process.

The Delphi process was structured and transparent adding validity to the results. The facilitator was fundamental for the successful implementation of the Delphi, ensuring the effective participation of panellists and managing the group process toward agreement. In addition, the use of a web platform to deliver the survey and to follow-up the process increased the efficiency of the procedures. This type of study is very sensitive to how a question or statement is formulated. To address bias, one of the potential limitations of Delphi studies [82], care was taken to provide feedback in a neutral way, so as not to influence the panellist response. The anonymity of participants, a commonly advantage of Delphi studies, was assured.

The high levels of participation observed in this study shows the potential to develop a shared understanding about what is important for appraising population health, and to contribute for a sense of common purpose about which indicators are critical for policies in line with improving health and decreasing health inequities. The final set of indicators is valid and consistent with a range of important dimensions related to health, covering well-known social determinants, such as social, economic, demographic, lifestyles and environmental conditions, and also dimensions within the traditional scope of health, namely the healthcare services and health outcomes.

However, the external validity of the findings in this study may be limited because many of the indicators will tend to reflect what the EURO-HEALTHY panel of experts and stakeholders consider relevant in relation to the European regional context. It is likely that certain indicators would be considered more or less relevant depending on the panel and for distinct contexts. This study should be seen as a starting point for discussing population health indicator sets to be used in European statistical systems, adapted to local contexts and involving local panels of stakeholders, and on identifying research priorities relating to population health measurement and framing data collection and monitoring needs.

Other potential limitation is linked to the Delphi panel composition and the risk of researcher bias, since it included a higher number of experts (51 researchers) comparing with the invited stakeholders (30). Practically speaking, the involvement of stakeholders, including policy-makers is directly dependent on the resources available to bring together individuals from different levels of decision and from different countries and regions. In our study, project’ researchers indicated those who should make up the panel based on their interest in achieving the study purpose, thus considering the national and regional networks. This led to a potential impact on the regional representativeness, since not all 28 EU Member States were represented in the panel. Establishing stakeholder representativeness can often prove difficult in Delphi studies, given limits on time, resources and other. The role of stakeholders and its participation in decision-making studies is increasing, and thus further research in this topic should be enhanced, namely to inform the dimension and composition of Delphi panels according to the topic and objective, in order to assure the representativeness of their values and goals [83].

### Further research

Evaluating population health is challenging, considering that health is multidimensional and many authors use a wide range of indicators including health determinants and health outcomes. Involving experts and stakeholders from different backgrounds, fields of expertise and geographical locations adds diversity of points of view and validates the holistic perspective on health. Consequently, further research should be carried out to tailor the participatory approach, methods and results to varying contexts. More studies are desirable for testing this framework at distinct geographic scales (e.g. local) and locations (e.g. Africa, Latin America) to compare different perspectives. Thus, it is important to understand the factors that drive the experts’ different opinions regarding the relevance of indicators to appraise population health (namely related with the expert background and geographical context) and if the differences in opinion are systematic or random.

As a result of the successful application of the Delphi on the selection of indicators, the use of Web Delphi was extended and integrated with the MACBETH approach to collect panellist’s judgements in the multicriteria modelling of the PHI. Further work will also be undertaken on the relevance of using the Delphi technique and on the implications of using this type of participatory approach on policy development regarding population health.

## Conclusions

This study provides a comprehensive and sound analysis of the application of a Delphi process to inform the selection of population health indicators for the context of Europe at regional level. Our study adds to literature namely by: 1) from the practical viewpoint, promoting a consensus among a range of stakeholders from different areas of knowledge and geographies on which indicators are mostly relevant for appraising population health at European regional level; 2) from a methodological perspective, by proposing participatory methods and applying statistical analysis of responses that can be used in other health contexts iii) and from the technology side, by presenting an innovative web-platform that enables the use of participatory processes and its monitoring.

Furthermore, this study highlighted the usefulness of relevant stakeholder involvement in an indicator selection process, whilst showing the different views and perceptions that exist. The findings from the study can inform future research on population health measurement since it focuses the importance of, further to the use of scientific evidence, the need to account for the points of view of experts and stakeholders from different backgrounds and expertise (including Health and Social Sciences and Humanities).

## Additional files

Additional file 1: List of potential indicators, grouped by area of concern and dimension, identified in a literature review prior to the Web Delphi survey. (DOCX 21 kb)

Additional file 2: Web Delphi survey screens illustrating the implementation and monitoring of the Delphi process on the web platform. (DOCX 6129 kb)

Additional file 3: Results of the Delphi process with aggregated responses from the panel. (DOCX 46 kb)

## Abbreviations

A: Agree
BTE: Built environment
D: Disagree
DC: Demographic change
EHESS: Environmental health, ecological systems and sustainability
EHS: Economics and health systems
ESE: Economic and social environment
ESMPH: Epidemiology, social medicine and public health
HCS: Healthcare services
HGDS: Health geography, demography and sociology
HO: Health outcomes
IRR: Inter-Rater Reliability
LHB: Lifestyle and Health behaviours
MANOVA: Multivariate Analyses Of Variance
NAD: Neither agree nor disagree
PHE: Physical environment
PHI: Population Health Index
SA: Strongly agree
SD: Strongly disagree
