# Supplementary material for: Indicators for evaluating European population health: a Delphi selection process
Source: BMC Public Health. 2018 Apr 27;18:557. doi: 10.1186/s12889-018-5463-0 (PMC5922019; doi:10.1186/s12889-018-5463-0)
Supplement: Supplementary file 1 — List of potential indicators, grouped by area of concern and dimension, identified in a literature review prior to the Web Delphi survey. (DOCX 21 kb) [file 12889_2018_5463_MOESM1_ESM.docx]

**Additional file 1.** List of potential indicators, grouped by area of concern and dimension, identified in a literature review prior to the Web Delphi survey.

| **No.** | **Indicator** | **Dimension** |
| --- | --- | --- |
| **ECONOMIC AND SOCIAL ENVIRONMENT** | |  |
| It1 | Unemployment rate (%) | Employment |
| It2 | Youth unemployment rate (%) | Employment |
| It3 | Long-term unemployment rate -12 months and more (%) | Employment |
| It4 | Unemployment gender ratio | Employment |
| It5 | Gross Domestic Product, per capita in Purchasing Power Standards (PPS) | Income & living conditions |
| It6 | Disposable income of private households, in power consumption standards (PPCS) | Income & living conditions |
| It7 | People at risk of poverty or social exclusion (%) | Income & living conditions |
| It8 | People living in households with very low work intensity - aged 0 to 59 years (%) | Income & living conditions |
| It9 | Severe material deprivation rate | Income & living conditions |
| It10 | Gini Coefficient | Income & living conditions |
| It11 | Disposable income ratio (S80/S20) | Income & living conditions |
| It12 | Beneficiaries of disability pension | Social protection |
| It13 | Social protection benefits - social exclusion (% of total benefits) | Social protection |
| It14 | Expenditure on social protection benefits (% of GDP) | Social protection |
| It15 | Expenditure on care for elderly (% of GDP) | Social protection |
| It16 | Population aged 25-64 with lower secondary education attainment (%) | Education |
| It17 | Population aged 25-64 with tertiary education attainment (%) | Education |
| It18 | Early leavers from education and training (%) | Education |
| It19 | Households with access to the internet at home (%) | ICT access and use |
| It20 | Voter turnout in national elections (%) | Governance |
| It21 | Voter turnout in EU parliamentary elections (%) | Governance |
| It22 | Satisfaction with democracy in one's country and in the EU | Governance |
| It23 | Population who reported crime, violence or vandalism in the area of residence | Security |
| **DEMOGRAPHIC CHANGE** | |  |
| It24 | Immigrants from EU-28 countries (%) | Migration |
| It25 | Immigrants from Non EU28-countries (%) | Migration |
| It26 | Immigrants at risk of poverty or social exclusion, born in EU28 countries | Migration |
| It27 | Immigrants at risk of poverty or social exclusion, born in non EU28 countries | Migration |
| It28 | Average crude rate of net migration (including statistical adjustment) | Migration |
| It29 | At-risk-of-poverty rate of older people - aged 65 years or over (%) | Ageing |
| It30 | Ageing index | Ageing |
| It31 | Age dependency ratio | Ageing |
| It32 | Crude birth rate | Population Change |
| It33 | Population change between two consecutive years | Population Change |
| It34 | Fertility rate | Population Change |
| **LIFESTYLES AND HEALTH BEHAVIOURS** | |  |
| It35 | Adults who are obese (%) | Lifestyle & Health Behaviours |
| It36 | Regular daily smokers in the population - aged 15 and over (%) | Lifestyle & Health Behaviours |
| It37 | Daily smokers - aged 15 and over (%) | Lifestyle & Health Behaviours |
| It38 | Number of cigarettes smoked per day - daily smokers | Lifestyle & Health Behaviours |
| It39 | Pure alcohol consumption - aged 15 and over (litres per capita) | Lifestyle & Health Behaviours |
| It40 | Population engaged in vigorous or moderate physical activity on 2 or more days a week | Lifestyle & Health Behaviours |
| It41 | Average amount of fruits and vegetables available per person per year | Lifestyle & Health Behaviours |
| It42 | Average number of calories available per person per day | Lifestyle & Health Behaviours |
| It43 | Protein available per person per day | Lifestyle & Health Behaviours |
| It44 | Live births by mothers under age of 20 (%) | Lifestyle & Health Behaviours |
| **PHYSICAL ENVIRONMENT** | |  |
| It45 | Annual mean concentrations of Particulate Matter - PM2.5 (ug/m3) | Pollution |
| It46 | Annual mean concentrations of Particulate Matter - PM10 (ug/m3) | Pollution |
| It47 | Annual mean ozone concentrations | Pollution |
| It48 | Annual mean Nitrogen Dioxide concentration | Pollution |
| It49 | Annual mean Sulphur Dioxide concentration | Pollution |
| It50 | Transport emissions of air pollutants | Pollution |
| It51 | Number of people exposed to traffic noise | Pollution |
| It52 | Population who reported to be affected by noise from neighbours or from the street in the area of residence | Pollution |
| It53 | Population who reported pollution, grime or other environmental problems in the area of residence | Pollution |
| It54 | Particulate Matter (PM2.5 and PM10) emissions by industry and households | Pollution |
| It55 | Particulate Matter (PM2.5 and PM10) emissions from main source sectors (tonne) | Pollution |
| It56 | Greenhouse Gas Emissions (thousand tonnes) - CO2 equivalent | Pollution |
| It57 | Urban population exposure to air pollution by particulate matter PM10 | Pollution |
| It58 | Urban population exposure to air pollution by ozone (Micrograms per cubic metre day) | Pollution |
| It59 | Biochemical oxygen demand in rivers (mg O2/L) | Pollution |
| It60 | Contaminated sites and other land use indicators | Pollution |
| It61 | Number of high temperature days | Extreme weather events |
| It62 | Number of low temperature days | Extreme weather events |
| It63 | Number of warm nights | Extreme weather events |
| It64 | Number of heatwaves | Extreme weather events |
| It65 | Heating degree days | Extreme weather events |
| It66 | Number of people affected by flooding | Extreme weather events |
| **BUILT ENVIRONMENT** | |  |
| It67 | Average number of rooms per person | Housing conditions |
| It68 | Children living in homes with problems of dampness | Housing conditions |
| It69 | Population living in a dwelling with a leaking roof, damp walls, floors or foundation, or rot in window frames of floor | Housing conditions |
| It70 | Population having neither a bath, nor a shower, nor indoor flushing toilet in their household | Housing conditions |
| It71 | Population who reported inability to keep home adequately warm | Housing conditions |
| It72 | Ventilation rates in EU dwellings | Housing conditions |
| It73 | Population density | Land use and transport |
| It74 | Passenger transport volume, by car | Land use and transport |
| It75 | Water Exploitation Index | Water and sanitation |
| It76 | Population connected to public water supply | Water and sanitation |
| It77 | Drinking water quality: microbiological non-compliance | Water and sanitation |
| It78 | Drinking water quality: chemical non-compliance | Water and sanitation |
| It79 | Population connected to wastewater treatment | Water and sanitation |
| It80 | Waste generation - Total waste generation | Waste management |
| It81 | Waste treatment - Total waste treatment | Waste management |
| It82 | Municipal waste treatment, by material recycling | Waste management |
| It83 | Pedestrian and bicyclist deaths | Road Safety |
| It84 | Injury rate due to road traffic accidents in the total population | Road Safety |
| It85 | Victims in road accidents - injured and killed | Road Safety |
| It86 | Fatality rate due to road traffic accidents | Road Safety |
| **HEALTHCARE SERVICES** | |  |
| It87 | Health personnel (General practitioners) per 100.000 inhabitants | Healthcare Resources |
| It88 | Curative care beds in hospitals per 100.000 inhabitants | Healthcare Resources |
| It89 | Long-term care beds in nursing and residential care facilities per 100.000 inhabitants | Healthcare Resources |
| It90 | Medical doctors per 100.000 inhabitants | Healthcare Resources |
| It91 | Health personnel (medical doctors, nurses and midwives, dentists, pharmacists and physiotherapists) per 100.000 inhabitants | Healthcare Resources |
| It92 | Medical Technology (e.g. mammography, PET scanners, computed tomography scanners) per 100.000 inhabitants | Healthcare Resources |
| It93 | Population living to more than 45 minutes of a maternity | Healthcare Access |
| It94 | Population living to more than 60 minutes of Urgency/Emergency unit | Healthcare Access |
| It95 | Hospital discharges due to diabetes, hypertension and asthma per 100.000 inhabitants | Healthcare utilization |
| It96 | Health care expenditure by all financing agents per inhabitant, in Purchasing Power Standards (PPS) | Health Expenditure |
| It97 | Health care expenditure by private household out-of-pocket expenditure per inhabitant, in Purchasing Power Standards (PPS) | Health Expenditure |
| It98 | Governmental (except social security funds) expenditure in providers of health care in health care per capita, in Purchasing Power Standards (PPS) | Health Expenditure |
| **HEALTH OUTCOMES** | |  |
| It99 | Life expectancy at birth | Length of life (Mortality) |
| It100 | Sex ratio of life expectancy at birth | Length of life (Mortality) |
| It101 | Life expectancy at 65 years old | Length of life (Mortality) |
| It102 | Infant mortality per 1000 live births | Length of life (Mortality) |
| It103 | Perinatal mortality | Length of life (Mortality) |
| It104 | Neonatal mortality | Length of life (Mortality) |
| It105 | Late foetal mortality | Length of life (Mortality) |
| It106 | Premature mortality, SDR per 100.000 inhabitants | Length of life (Mortality) |
| It107 | Sex ratio of premature mortality | Length of life (Mortality) |
| It108 | Amenable deaths to health care, SDR per 100.000 inhabitants | Length of life (Mortality) |
| It109 | Preventable deaths, SDR per 100.000 inhabitants | Length of life (Mortality) |
| It110 | Sex ratio of amenable mortality | Length of life (Mortality) |
| It111 | Sex ratio of preventable mortality | Length of life (Mortality) |
| It112 | Deaths related to dementias including Alzheimer's disease, SDR per 100.000 inhabitants | Length of life (Mortality) |
| It113 | Deaths related to infectious disease, SDR per 100.000 inhabitants | Length of life (Mortality) |
| It114 | Deaths related to land transport accidents, SDR per 100.000 inhabitants | Length of life (Mortality) |
| It115 | Deaths from diseases of the circulatory system, SDR per 100.000 inhabitants | Length of life (Mortality) |
| It116 | Deaths from diseases of the respiratory system, SDR per 100.000 inhabitants | Length of life (Mortality) |
| It117 | Deaths from cancer (malignant neoplasms), SDR per 100.000 inhabitants | Length of life (Mortality) |
| It118 | Death from ischaemic heart disease, SDR per 100.000 inhabitants | Length of life (Mortality) |
| It119 | Deaths from cerebrovascular diseases, SDR per 100.000 inhabitants | Length of life (Mortality) |
| It120 | Deaths from colorectal cancer, SDR per 100.000 inhabitants | Length of life (Mortality) |
| It121 | Deaths from larynx, trachea, bronchus and lung cancer, SDR per 100.000 inhabitants | Length of life (Mortality) |
| It122 | Deaths from breast cancer, SDR per 100.000 inhabitants | Length of life (Mortality) |
| It123 | Deaths from prostate cancer, SDR per 100.000 inhabitants | Length of life (Mortality) |
| It124 | People who reported having a long-standing illness or health problem | Quality of life (Morbidity) |
| It125 | Self-perceived health less than good | Quality of life (Morbidity) |
| It126 | Self-perceived long-standing limitations in usual activities due to health problem | Quality of life (Morbidity) |
| It127 | Self-reported unmet needs for medical examination | Quality of life (Morbidity) |
| It128 | Age-standardized Disability-Adjusted Life Year (DALY) rate | Quality of life (Morbidity) |
| It129 | Low birth-weight | Quality of life (Morbidity) |
| It130 | Preterm birth | Quality of life (Morbidity) |
