# Supplementary material for: Indicators for evaluating European population health: a Delphi selection process
Source: BMC Public Health. 2018 Apr 27;18:557. doi: 10.1186/s12889-018-5463-0 (PMC5922019; doi:10.1186/s12889-018-5463-0)
Supplement: Supplementary file 2 — Web Delphi survey screens illustrating the implementation and monitoring of the Delphi process on the web platform. (DOCX 6129 kb) [file 12889_2018_5463_MOESM2_ESM.docx]

**Additional file 2.** Web Delphi survey screens illustrating the implementation and monitoring of the Delphi process on the web platform. Source: BanaConsulting, Lda


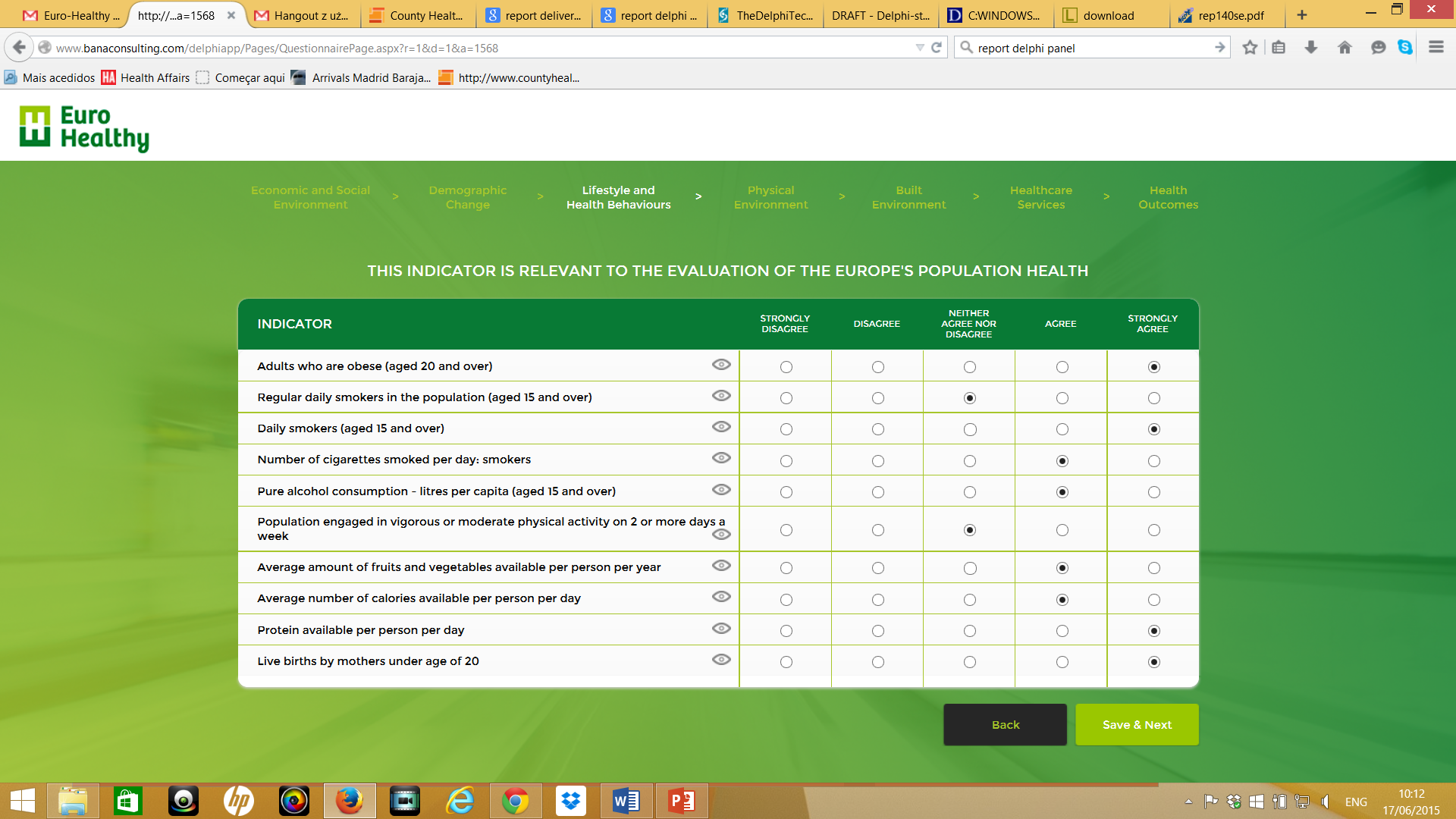

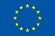


EURO-HEALTHY project has received funding from the European Union’s Horizon 2020 research and innovation programme under Grant Agreement No 643398.

(A) Web Delphi survey questionnaire screen for the assessment. In each area of concern, panellists were asked to indicate the level of agreement and disagreement regarding the relevance of indicators, in a 5-level Likert scale.


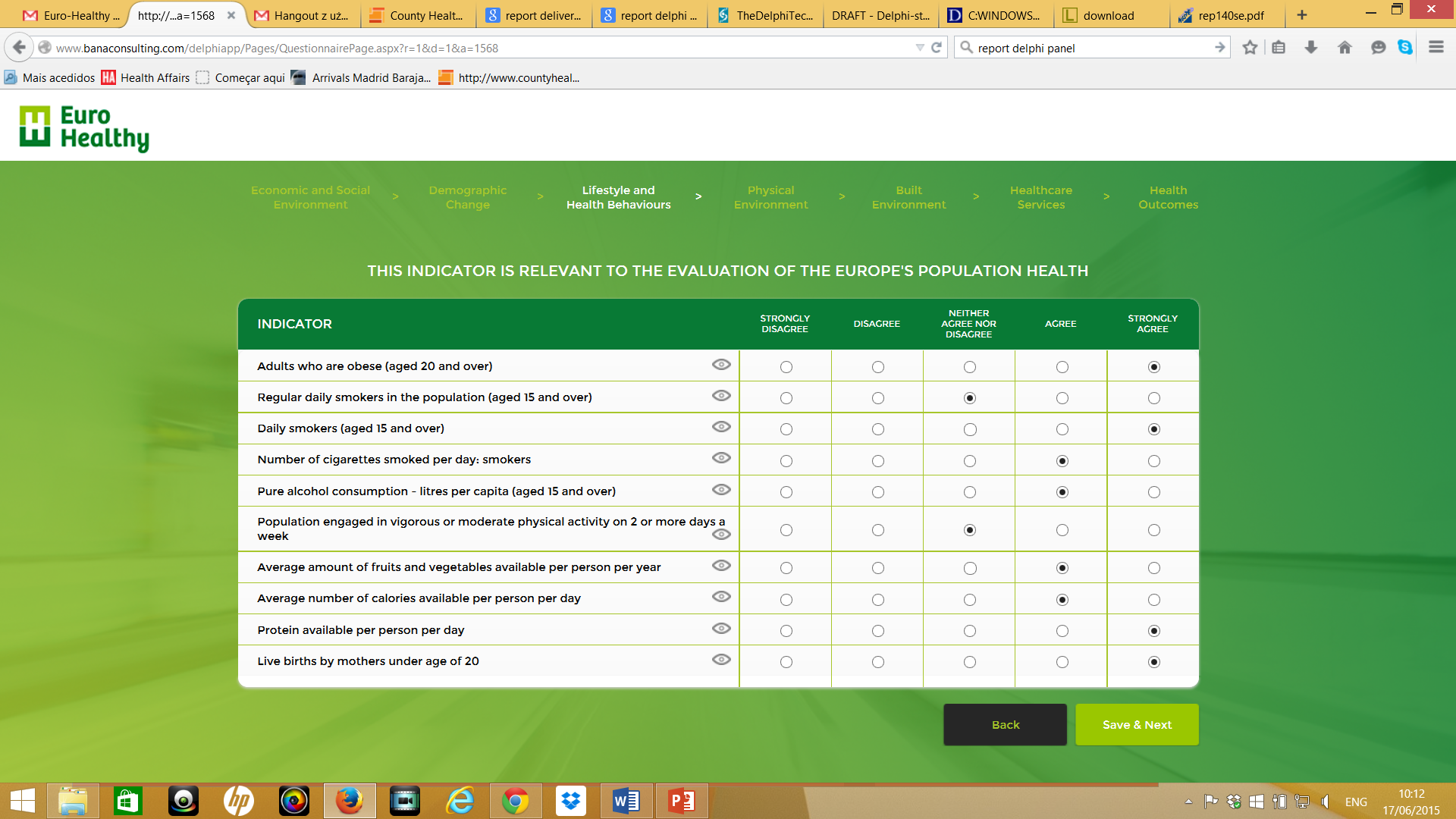

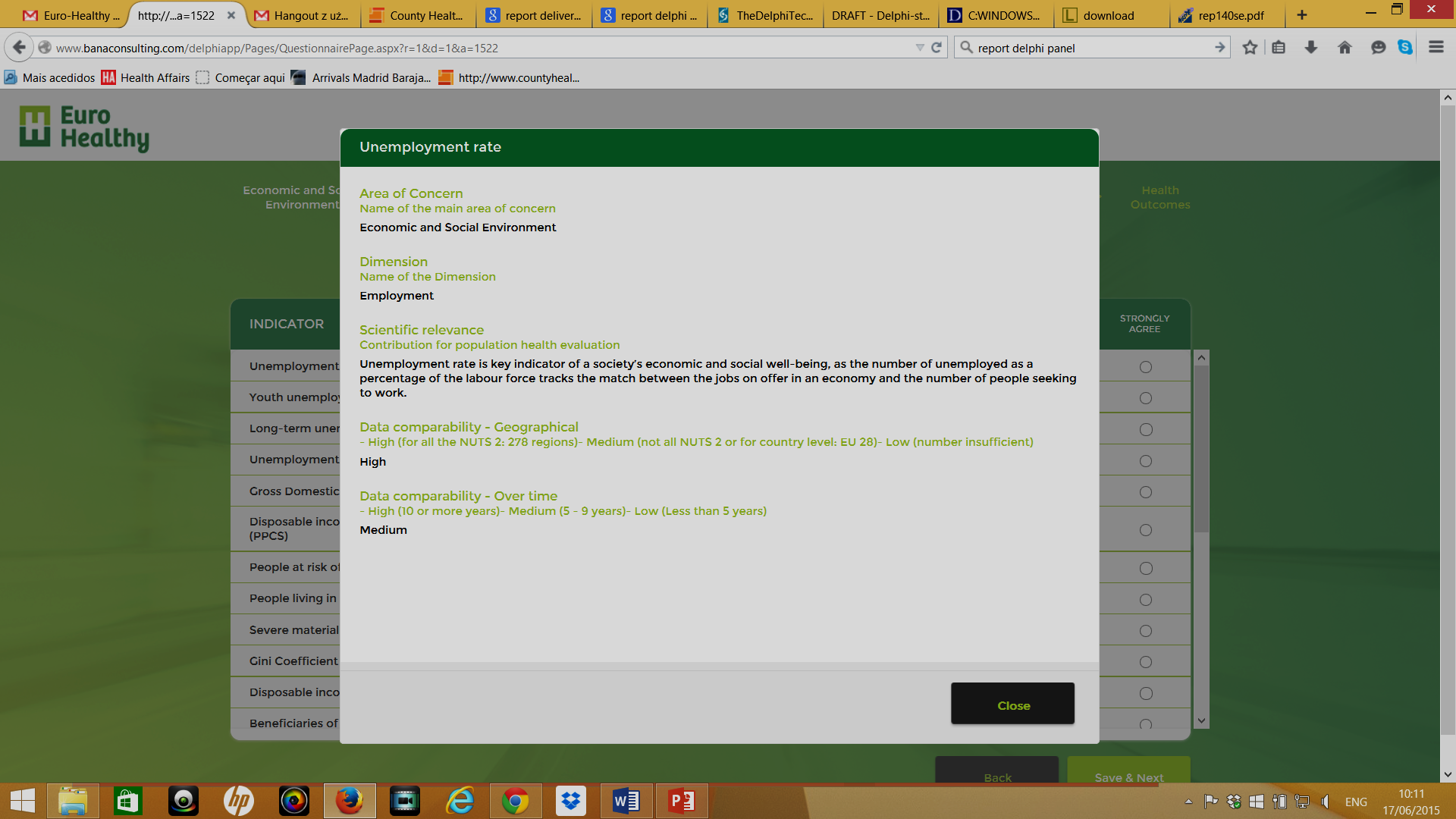

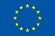


EURO-HEALTHY project has received funding from the European Union’s Horizon 2020 research and innovation programme under Grant Agreement No 643398.

B) Web Delphi survey questionnaire screen with indicator information.


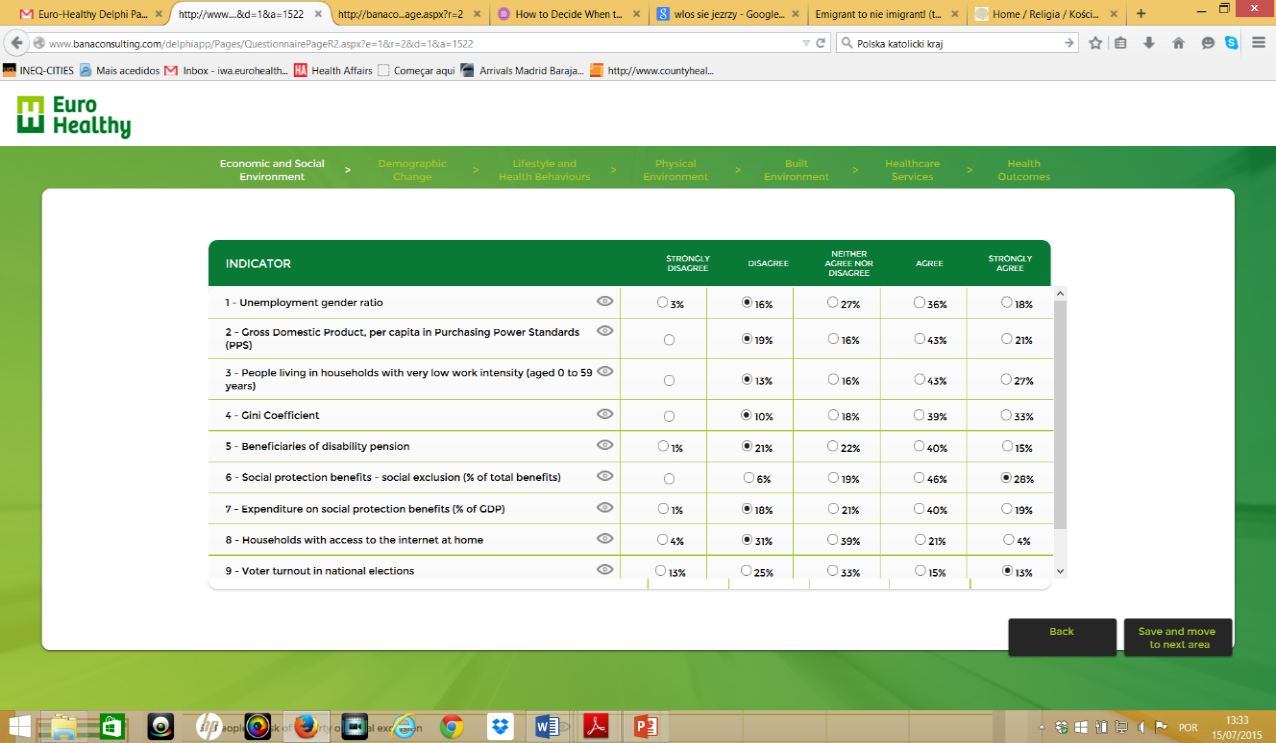

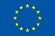


EURO-HEALTHY project has received funding from the European Union’s Horizon 2020 research and innovation programme under Grant Agreement No 643398.

(C) Web Delphi survey questionnaire feedback screen.
