# Supplementary material for: Indicators for evaluating European population health: a Delphi selection process
Source: BMC Public Health. 2018 Apr 27;18:557. doi: 10.1186/s12889-018-5463-0 (PMC5922019; doi:10.1186/s12889-018-5463-0)
Supplement: Supplementary file 3 — Results of the Delphi process with aggregated responses from the panel. (DOCX 46 kb) [file 12889_2018_5463_MOESM3_ESM.docx]

**Additional file 3.** Results of the Delphi process with aggregated responses from the panel.

| **No.** | **Round 1** | | | | | | | | | | **Round 2** | | | | | | | | | | **Round 3** | | | | | | | | | | **Group majority decision rule** |
| --- | --- | --- | --- | --- | --- | --- | --- | --- | --- | --- | --- | --- | --- | --- | --- | --- | --- | --- | --- | --- | --- | --- | --- | --- | --- | --- | --- | --- | --- | --- | --- |
|  | **Aggregated responses (%)** | | | | | **M** | **Me** | **CV** | **IQR** | ***Pi*** | **Aggregated responses (%)** | | | | | **M** | **Me** | **CV** | **IQR** | ***Pi*** | **Aggregated responses (%)** | | | | | **M** | **Me** | **CV** | **IQR** | ***Pi*** |  |
|  | **SD  (1)** | **D (2)** | **NAD (3)** | **A (4)** | **SA (5)** |  |  |  |  |  | **SD  (1)** | **D (2)** | **NAD (3)** | **A (4)** | **SA (5)** |  |  |  |  |  | **SD  (1)** | **D (2)** | **NAD (3)** | **A (4)** | **SA (5)** |  |  |  |  |  |  |
| It1 | 1.4 | 1.4 | 2.8 | 30.6 | 63.9 | 4.5 | 5.0 | 0.2 | 1.0 | 0.5 | - | - | - | - | - | - | - | - | - | - | - | - | - | - | - | - | - | - | - | - | A1-A |
| It2 | 1.4 | 4.2 | 12.5 | 41.7 | 40.3 | 4.2 | 4.0 | 0.2 | 1.0 | 0.3 | 0.0 | 3.0 | 9.0 | 49.3 | 38.8 | 4.2 | 4.0 | 0.2 | 1.0 | 0.4 | - | - | - | - | - | - | - | - | - | - | A2-B |
| It3 | 1.4 | 4.2 | 4.2 | 25.0 | 65.3 | 4.5 | 5.0 | 0.2 | 1.0 | 0.5 | - | - | - | - | - | - | - | - | - | - | - | - | - | - | - | - | - | - | - | - | A1-A |
| It4 | 4.2 | 9.7 | 25.0 | 37.5 | 23.6 | 3.7 | 4.0 | 0.3 | 1.0 | 0.3 | 3.0 | 16.4 | 26.9 | 35.8 | 17.9 | 3.5 | 4.0 | 0.3 | 1.0 | 0.2 | 1.6 | 17.2 | 26.6 | 39.1 | 15.6 | 3.5 | 4.0 | 0.3 | 1.0 | 0.3 | NC |
| It5 | 1.4 | 9.7 | 18.1 | 40.3 | 30.6 | 3.9 | 4.0 | 0.3 | 2.0 | 0.3 | 0.0 | 19.4 | 16.4 | 43.3 | 20.9 | 3.7 | 4.0 | 0.3 | 1.0 | 0.3 | 3.1 | 14.1 | 14.1 | 51.6 | 17.2 | 3.7 | 4.0 | 0.3 | 1.0 | 0.3 | NC |
| It6 | 1.4 | 2.8 | 15.3 | 44.4 | 36.1 | 4.1 | 4.0 | 0.2 | 1.0 | 0.3 | 0.0 | 3.0 | 11.9 | 50.7 | 34.3 | 4.2 | 4.0 | 0.2 | 1.0 | 0.4 | - | - | - | - | - | - | - | - | - | - | A2-B |
| It7 | 1.4 | 1.4 | 4.2 | 20.8 | 72.2 | 4.6 | 5.0 | 0.2 | 1.0 | 0.6 | - | - | - | - | - | - | - | - | - | - | - | - | - | - | - | - | - | - | - | - | A1-A |
| It8 | 1.4 | 5.6 | 20.8 | 43.1 | 29.2 | 3.9 | 4.0 | 0.2 | 2.0 | 0.3 | 0.0 | 13.4 | 16.4 | 43.3 | 26.9 | 3.8 | 4.0 | 0.3 | 2.0 | 0.3 | 6.3 | 14.1 | 10.9 | 51.6 | 17.2 | 3.6 | 4.0 | 0.3 | 1.0 | 0.3 | NC |
| It9 | 2.8 | 2.8 | 4.2 | 38.9 | 51.4 | 4.3 | 5.0 | 0.2 | 1.0 | 0.4 | - | - | - | - | - | - | - | - | - | - | - | - | - | - | - | - | - | - | - | - | A1-A |
| It10 | 0.0 | 4.2 | 20.8 | 37.5 | 37.5 | 4.1 | 4.0 | 0.2 | 1.3 | 0.3 | 0.0 | 10.4 | 17.9 | 38.8 | 32.8 | 4.0 | 3.9 | 0.2 | 2.0 | 0.3 | 6.3 | 9.4 | 12.5 | 46.9 | 25.0 | 3.8 | 4.0 | 0.3 | 1.3 | 0.3 | NC |
| It11 | 2.8 | 2.8 | 20.8 | 38.9 | 34.7 | 4.0 | 4.0 | 0.2 | 2.0 | 0.3 | 1.5 | 4.5 | 16.4 | 34.3 | 43.3 | 4.0 | 4.1 | 0.2 | 1.0 | 0.3 |  |  |  |  |  |  |  |  |  |  | A2-B |
| It12 | 2.8 | 12.5 | 27.8 | 41.7 | 15.3 | 3.5 | 4.0 | 0.3 | 1.0 | 0.3 | 1.5 | 20.9 | 22.4 | 40.3 | 14.9 | 4.0 | 3.5 | 0.3 | 1.0 | 0.3 | 1.6 | 25.0 | 14.1 | 50.0 | 9.4 | 3.4 | 4.0 | 0.3 | 2.0 | 0.3 | NC |
| It13 | 1.4 | 5.6 | 26.4 | 45.8 | 20.8 | 3.8 | 4.0 | 0.2 | 1.0 | 0.3 | 0.0 | 6.0 | 19.4 | 46.3 | 28.4 | 4.0 | 4.0 | 0.2 | 1.5 | 0.3 | 1.6 | 6.3 | 18.8 | 48.4 | 25.0 | 3.9 | 4.0 | 0.2 | 1.3 | 0.3 | NC |
| It14 | 2.8 | 9.7 | 27.8 | 37.5 | 22.2 | 3.7 | 4.0 | 0.3 | 1.0 | 0.3 | 1.5 | 17.9 | 20.9 | 40.3 | 19.4 | 4.0 | 3.6 | 0.3 | 1.0 | 0.3 | 0.0 | 20.3 | 18.8 | 43.8 | 17.2 | 3.6 | 4.0 | 0.3 | 1.0 | 0.3 | NC |
| It15 | 1.4 | 6.9 | 23.6 | 45.8 | 22.2 | 3.8 | 4.0 | 0.2 | 1.0 | 0.3 | 0.0 | 9.0 | 14.9 | 47.8 | 28.4 | 4.0 | 4.0 | 0.2 | 1.0 | 0.3 | - | - | - | - | - | - | - | - | - | - | A2-B |
| It16 | 1.4 | 6.9 | 12.5 | 41.7 | 37.5 | 4.1 | 4.0 | 0.2 | 1.0 | 0.3 | 0.0 | 14.9 | 7.5 | 46.3 | 31.3 | 4.0 | 3.9 | 0.3 | 1.0 | 0.3 | - | - | - | - | - | - | - | - | - | - | A2-B |
| It17 | 1.4 | 8.3 | 15.3 | 44.4 | 30.6 | 3.9 | 4.0 | 0.2 | 1.3 | 0.3 | 0.0 | 9.0 | 10.4 | 52.2 | 28.4 | 4.0 | 4.0 | 0.2 | 1.0 | 0.4 | - | - | - | - | - | - | - | - | - | - | A2-B |
| It18 | 1.4 | 6.9 | 22.2 | 34.7 | 34.7 | 3.9 | 4.0 | 0.3 | 2.0 | 0.3 | 0.0 | 9.0 | 13.4 | 41.8 | 35.8 | 4.0 | 4.0 | 0.2 | 1.0 | 0.3 | - | - | - | - | - | - | - | - | - | - | A2-B |
| It19 | 5.6 | 23.6 | 40.3 | 19.4 | 11.1 | 3.1 | 3.0 | 0.3 | 2.0 | 0.3 | 4.5 | 31.3 | 38.8 | 20.9 | 4.5 | 3.0 | 2.9 | 0.3 | 1.5 | 0.3 | 4.7 | 39.1 | 37.5 | 17.2 | 1.6 | 2.7 | 3.0 | 0.3 | 1.0 | 0.3 | NC |
| It20 | 12.5 | 26.4 | 31.9 | 16.7 | 12.5 | 2.9 | 3.0 | 0.4 | 2.0 | 0.2 | 13.4 | 25.4 | 32.8 | 14.9 | 13.4 | 2.9 | 3.0 | 0.4 | 2.0 | 0.2 | 14.1 | 29.7 | 35.9 | 9.4 | 10.9 | 2.7 | 3.0 | 0.4 | 1.0 | 0.2 | NC |
| It21 | 16.7 | 38.9 | 30.6 | 6.9 | 6.9 | 2.5 | 2.0 | 0.4 | 1.0 | 0.3 | - | - | - | - | - | - | - | - | - | - | - | - | - | - | - | - | - | - | - | - | R1 |
| It22 | 8.3 | 29.2 | 31.9 | 26.4 | 4.2 | 2.9 | 3.0 | 0.4 | 2.0 | 0.3 | 9.0 | 32.8 | 35.8 | 20.9 | 1.5 | 2.7 | 3.0 | 0.3 | 1.0 | 0.3 | 9.4 | 35.9 | 37.5 | 17.2 | 0.0 | 2.6 | 3.0 | 0.3 | 1.0 | 0.3 | NC |
| It23 | 1.4 | 9.7 | 9.7 | 50.0 | 29.2 | 4.0 | 4.0 | 0.2 | 1.0 | 0.3 | 0.0 | 6.0 | 7.5 | 50.7 | 35.8 | 4.2 | 4.0 | 0.2 | 1.0 | 0.4 |  |  |  |  |  |  |  |  |  |  | A2-B |
| It24 | 8.3 | 27.8 | 30.6 | 29.2 | 4.2 | 2.9 | 3.0 | 0.4 | 2.0 | 0.3 | 14.9 | 28.4 | 28.4 | 26.9 | 1.5 | 2.7 | 3.0 | 0.4 | 2.0 | 0.2 | 15.6 | 34.4 | 26.6 | 21.9 | 1.6 | 2.6 | 2.5 | 0.4 | 1.0 | 0.2 | NC |
| It25 | 2.8 | 12.5 | 29.2 | 37.5 | 18.1 | 3.6 | 4.0 | 0.3 | 1.0 | 0.3 | 7.5 | 11.9 | 25.4 | 43.3 | 11.9 | 3.4 | 4.0 | 0.3 | 1.0 | 0.3 | 6.3 | 9.4 | 23.4 | 51.6 | 9.4 | 3.5 | 4.0 | 0.3 | 1.0 | 0.3 | NC |
| It26 | 4.2 | 4.2 | 20.8 | 48.6 | 22.2 | 3.8 | 4.0 | 0.3 | 1.0 | 0.3 | 1.5 | 4.5 | 7.5 | 61.2 | 25.4 | 4.0 | 4.0 | 0.2 | 0.5 | 0.4 | - | - | - | - | - | - | - | - | - | - | A2-B |
| It27 | 2.8 | 2.8 | 16.7 | 48.6 | 29.2 | 4.0 | 4.0 | 0.2 | 1.0 | 0.3 | 0.0 | 1.5 | 7.5 | 58.2 | 32.8 | 4.2 | 4.0 | 0.2 | 1.0 | 0.4 | - | - | - | - | - | - | - | - | - | - | A2-B |
| It28 | 2.8 | 18.1 | 37.5 | 26.4 | 15.3 | 3.3 | 3.0 | 0.3 | 1.0 | 0.3 | 4.5 | 22.4 | 43.3 | 23.9 | 6.0 | 3.0 | 3.0 | 0.3 | 2.0 | 0.3 | 6.3 | 20 | 47 | 22 | 4.7 | 3 | 3 | 0 | 2 | 0 | NC |
| It29 | 1.4 | 1.4 | 11.1 | 47.2 | 38.9 | 4.2 | 4.0 | 0.2 | 1.0 | 0.4 | 0.0 | 1.5 | 4.5 | 50.7 | 43.3 | 4.4 | 4.0 | 0.1 | 1.0 | 0.4 | - | - | - | - | - | - | - | - | - | - | A2-B |
| It30 | 0.0 | 4.2 | 11.1 | 40.3 | 44.4 | 4.3 | 4.0 | 0.2 | 1.0 | 0.4 | 0.0 | 6.0 | 7.5 | 40.3 | 46.3 | 4.3 | 4.0 | 0.2 | 1.0 | 0.4 | - | - | - | - | - | - | - | - | - | - | A2-B |
| It31 | 0.0 | 5.6 | 19.4 | 41.7 | 33.3 | 4.0 | 4.0 | 0.2 | 1.3 | 0.3 | 0.0 | 7.5 | 11.9 | 50.7 | 29.9 | 4.0 | 4.0 | 0.2 | 1.0 | 0.4 | - | - | - | - | - | - | - | - | - | - | A2-B |
| It32 | 4.2 | 15.3 | 31.9 | 29.2 | 19.4 | 3.4 | 3.0 | 0.3 | 1.0 | 0.2 | 11.9 | 14.9 | 29.9 | 34.3 | 9.0 | 3.1 | 3.0 | 0.4 | 2.0 | 0.2 | 9.4 | 16 | 31 | 36 | 7.8 | 3 | 4 | 0 | 0.4 | 0 | NC |
| It33 | 5.6 | 16.7 | 48.6 | 15.3 | 13.9 | 3.2 | 3.0 | 0.3 | 1.0 | 0.3 | 13.4 | 19.4 | 47.8 | 14.9 | 4.5 | 2.8 | 3.0 | 0.4 | 1.0 | 0.3 | 14 | 22 | 48 | 13 | 3.1 | 3 | 4 | 0 | 0.3 | 0 | NC |
| It34 | 1.4 | 11.1 | 30.6 | 31.9 | 25.0 | 3.7 | 4.0 | 0.3 | 1.3 | 0.3 | 1.5 | 13.4 | 25.4 | 34.3 | 25.4 | 3.7 | 4.0 | 0.3 | 1.5 | 0.3 | 1.6 | 11 | 27 | 39 | 22 | 4 | 4 | 0 | 0.3 | 0 | NC |
| It35 | 0.0 | 1.4 | 1.4 | 25.0 | 72.2 | 4.7 | 5.0 | 0.1 | 1.0 | 0.6 | - | - | - | - | - | - | - | - | - | - | - | - | - | - | - | - | - | - | - | - | A1-A |
| It36 | 2.8 | 4.2 | 4.2 | 22.2 | 66.7 | 4.5 | 5.0 | 0.2 | 1.0 | 0.5 | - | - | - | - | - | - | - | - | - | - | - | - | - | - | - | - | - | - | - | - | A1-A |
| It37 | 2.8 | 6.9 | 5.6 | 27.8 | 56.9 | 4.3 | 5.0 | 0.2 | 1.0 | 0.4 | - | - | - | - | - | - | - | - | - | - | - | - | - | - | - | - | - | - | - | - | A1-A |
| It38 | 1.4 | 4.2 | 6.9 | 44.4 | 43.1 | 4.2 | 4.0 | 0.2 | 1.0 | 0.4 | 0.0 | 6.0 | 4.5 | 38.8 | 50.7 | 4.3 | 5.0 | 0.2 | 1.0 | 0.4 | - | - | - | - | - | - | - | - | - | - | A2-A |
| It39 | 0.0 | 0.0 | 4.2 | 43.1 | 52.8 | 4.5 | 5.0 | 0.1 | 1.0 | 0.5 | - | - | - | - | - | - | - | - | - | - | - | - | - | - | - | - | - | - | - | - | A1-A |
| It40 | 1.4 | 0.0 | 8.3 | 48.6 | 41.7 | 4.3 | 4.0 | 0.2 | 1.0 | 0.4 | 0.0 | 0.0 | 4.5 | 50.7 | 44.8 | 4.4 | 4.0 | 0.1 | 1.0 | 0.5 | - | - | - | - | - | - | - | - | - | - | A2-B |
| It41 | 0.0 | 0.0 | 13.9 | 55.6 | 30.6 | 4.2 | 4.0 | 0.2 | 1.0 | 0.4 | 0.0 | 0.0 | 11.9 | 55.2 | 32.8 | 4.2 | 4.0 | 0.2 | 1.0 | 0.4 | - | - | - | - | - | - | - | - | - | - | A2-B |
| It42 | 2.8 | 8.3 | 29.2 | 31.9 | 27.8 | 3.7 | 4.0 | 0.3 | 2.0 | 0.3 | 0.0 | 16.4 | 25.4 | 38.8 | 19.4 | 3.6 | 4.0 | 0.3 | 1.0 | 0.3 | 0.0 | 17.2 | 20.3 | 50.0 | 12.5 | 3.6 | 4.0 | 0.3 | 1.0 | 0.3 | NC |
| It43 | 2.8 | 6.9 | 48.6 | 26.4 | 15.3 | 3.4 | 3.0 | 0.3 | 1.0 | 0.3 | 0.0 | 19.4 | 49.3 | 25.4 | 6.0 | 3.2 | 3.0 | 0.3 | 1.0 | 0.3 | 0.0 | 25.0 | 53.1 | 17.2 | 4.7 | 3.0 | 3.0 | 0.3 | 0.3 | 0.4 | NC |
| It44 | 2.8 | 8.3 | 12.5 | 36.1 | 40.3 | 4.0 | 4.0 | 0.3 | 1.0 | 0.3 | 0.0 | 4.5 | 7.5 | 34.3 | 53.7 | 4.4 | 5.0 | 0.2 | 1.0 | 0.4 | - | - | - | - | - | - | - | - | - | - | A2-A |
| It45 | 0.0 | 0.0 | 22.2 | 31.9 | 45.8 | 4.2 | 4.0 | 0.2 | 1.0 | 0.4 | 0.0 | 0.0 | 13.4 | 34.3 | 52.2 | 4.4 | 5.0 | 0.2 | 1.0 | 0.4 | - | - | - | - | - | - | - | - | - | - | A2-A |
| It46 | 0.0 | 0.0 | 18.1 | 40.3 | 41.7 | 4.2 | 4.0 | 0.2 | 1.0 | 0.4 | 0.0 | 0.0 | 11.9 | 37.3 | 50.7 | 4.4 | 5.0 | 0.2 | 1.0 | 0.4 | - | - | - | - | - | - | - | - | - | - | A2-A |
| It47 | 2.8 | 1.4 | 26.4 | 34.7 | 34.7 | 4.0 | 4.0 | 0.2 | 2.0 | 0.3 | 7.5 | 1.5 | 20.9 | 37.3 | 32.8 | 3.9 | 4.0 | 0.3 | 2.0 | 0.3 | 7.8 | 3.1 | 17.2 | 43.8 | 28.1 | 3.8 | 4.0 | 0.3 | 2.0 | 0.3 | NC |
| It48 | 2.8 | 1.4 | 25.0 | 37.5 | 33.3 | 4.0 | 4.0 | 0.2 | 2.0 | 0.3 | 7.5 | 3.0 | 17.9 | 40.3 | 31.3 | 3.9 | 4.0 | 0.3 | 2.0 | 0.3 | 7.8 | 4.7 | 12.5 | 50.0 | 25.0 | 3.8 | 4.0 | 0.3 | 0.5 | 0.3 | NC |
| It49 | 2.8 | 0.0 | 27.8 | 37.5 | 31.9 | 4.0 | 4.0 | 0.2 | 2.0 | 0.3 | 7.5 | 3.0 | 20.9 | 40.3 | 28.4 | 3.8 | 4.0 | 0.3 | 2.0 | 0.3 | 7.8 | 6.3 | 14.1 | 48.4 | 23.4 | 3.7 | 4.0 | 0.3 | 1.0 | 0.3 | NC |
| It50 | 1.4 | 5.6 | 11.1 | 55.6 | 26.4 | 4.0 | 4.0 | 0.2 | 1.0 | 0.4 | 7.5 | 4.5 | 4.5 | 58.2 | 25.4 | 3.9 | 4.0 | 0.3 | 0.5 | 0.4 | - | - | - | - | - | - | - | - | - | - | A2-B |
| It51 | 2.8 | 6.9 | 20.8 | 56.9 | 12.5 | 3.7 | 4.0 | 0.2 | 1.0 | 0.4 | 7.5 | 3.0 | 14.9 | 64.2 | 10.4 | 3.7 | 4.0 | 0.3 | 0.5 | 0.4 | 6.3 | 3.1 | 10.9 | 73.4 | 6.3 | 3.7 | 4.0 | 0.2 | 0.0 | 0.6 | A3 |
| It52 | 0.0 | 13.9 | 33.3 | 40.3 | 12.5 | 3.5 | 4.0 | 0.3 | 1.0 | 0.3 | 1.5 | 17.9 | 23.9 | 46.3 | 10.4 | 3.5 | 4.0 | 0.3 | 1.0 | 0.3 | 3.1 | 14.1 | 26.6 | 50.0 | 6.3 | 3.4 | 4.0 | 0.3 | 1.0 | 0.3 | NC |
| It53 | 1.4 | 4.2 | 22.2 | 38.9 | 33.3 | 4.0 | 4.0 | 0.2 | 2.0 | 0.3 | 3.0 | 1.5 | 16.4 | 43.3 | 35.8 | 4.1 | 4.0 | 0.2 | 1.0 | 0.3 | - | - | - | - | - | - | - | - | - | - | A2-B |
| It54 | 2.8 | 2.8 | 26.4 | 36.1 | 31.9 | 3.9 | 4.0 | 0.2 | 2.0 | 0.3 | 1.5 | 13.4 | 19.4 | 37.3 | 28.4 | 3.8 | 4.0 | 0.3 | 2.0 | 0.3 | 1.6 | 15.6 | 17.2 | 43.8 | 21.9 | 3.7 | 4.0 | 0.3 | 1.0 | 0.3 | NC |
| It55 | 2.8 | 2.8 | 31.9 | 36.1 | 26.4 | 3.8 | 4.0 | 0.3 | 2.0 | 0.3 | 1.5 | 13.4 | 22.4 | 38.8 | 23.9 | 3.7 | 4.0 | 0.3 | 1.0 | 0.3 | 1.6 | 15.6 | 20.3 | 45.3 | 17.2 | 3.6 | 4.0 | 0.3 | 1.0 | 0.3 | NC |
| It56 | 2.8 | 5.6 | 31.9 | 40.3 | 19.4 | 3.7 | 4.0 | 0.3 | 1.0 | 0.3 | 9.0 | 7.5 | 22.4 | 46.3 | 14.9 | 3.5 | 4.0 | 0.3 | 1.0 | 0.3 | 6.3 | 10.9 | 14.1 | 57.8 | 10.9 | 3.6 | 4.0 | 0.3 | 1.0 | 0.4 | NC |
| It57 | 1.4 | 4.2 | 22.2 | 40.3 | 31.9 | 4.0 | 4.0 | 0.2 | 2.0 | 0.3 | 7.5 | 6.0 | 14.9 | 40.3 | 31.3 | 3.8 | 4.0 | 0.3 | 2.0 | 0.3 | 7.8 | 6.3 | 10.9 | 48.4 | 26.6 | 3.8 | 4.0 | 0.3 | 1.3 | 0.3 | NC |
| It58 | 2.8 | 5.6 | 27.8 | 41.7 | 22.2 | 3.8 | 4.0 | 0.3 | 1.0 | 0.3 | 7.5 | 7.5 | 20.9 | 43.3 | 20.9 | 3.6 | 4.0 | 0.3 | 1.0 | 0.3 | 7.8 | 7.8 | 15.6 | 53.1 | 15.6 | 3.6 | 4.0 | 0.3 | 1.0 | 0.3 | NC |
| It59 | 4.2 | 12.5 | 38.9 | 33.3 | 11.1 | 3.3 | 3.0 | 0.3 | 1.0 | 0.3 | 10.4 | 11.9 | 37.3 | 32.8 | 7.5 | 3.1 | 3.0 | 0.3 | 1.0 | 0.3 | 10.9 | 10.9 | 37.5 | 37.5 | 3.1 | 3.1 | 3.0 | 0.3 | 1.0 | 0.3 | NC |
| It60 | 1.4 | 2.8 | 16.7 | 45.8 | 33.3 | 4.1 | 4.0 | 0.2 | 1.0 | 0.3 | 1.5 | 1.5 | 14.9 | 47.8 | 34.3 | 4.1 | 4.0 | 0.2 | 1.0 | 0.4 | - | - | - | - | - | - | - | - | - | - | A2-B |
| It61 | 4.2 | 9.7 | 27.8 | 31.9 | 26.4 | 3.7 | 4.0 | 0.3 | 2.0 | 0.2 | 1.5 | 16.4 | 28.4 | 34.3 | 19.4 | 3.5 | 4.0 | 0.3 | 1.0 | 0.3 | 1.6 | 17.2 | 23.4 | 45.3 | 12.5 | 3.5 | 4.0 | 0.3 | 1.0 | 0.3 | NC |
| It62 | 5.6 | 11.1 | 31.9 | 33.3 | 18.1 | 3.5 | 4.0 | 0.3 | 1.0 | 0.3 | 9.0 | 11.9 | 31.3 | 29.9 | 17.9 | 3.4 | 3.0 | 0.4 | 1.0 | 0.2 | 9.4 | 12.5 | 29.7 | 35.9 | 12.5 | 3.3 | 3.0 | 0.3 | 1.0 | 0.2 | NC |
| It63 | 4.2 | 11.1 | 47.2 | 20.8 | 16.7 | 3.3 | 3.0 | 0.3 | 1.0 | 0.3 | 1.5 | 20.9 | 52.2 | 16.4 | 9.0 | 3.1 | 3.0 | 0.3 | 0.5 | 0.3 | 0.0 | 28.1 | 48.4 | 15.6 | 7.8 | 3.0 | 3.0 | 0.3 | 1.0 | 0.3 | NC |
| It64 | 2.8 | 8.3 | 26.4 | 26.4 | 36.1 | 3.8 | 4.0 | 0.3 | 2.0 | 0.3 | 1.5 | 17.9 | 29.9 | 22.4 | 28.4 | 3.6 | 4.0 | 0.3 | 2.0 | 0.2 | 0.0 | 23.4 | 26.6 | 23.4 | 26.6 | 3.5 | 3.5 | 0.3 | 2.0 | 0.2 | NC |
| It65 | 1.4 | 9.7 | 41.7 | 26.4 | 20.8 | 3.6 | 3.0 | 0.3 | 1.0 | 0.3 | 1.5 | 20.9 | 41.8 | 23.9 | 11.9 | 3.2 | 3.0 | 0.3 | 1.0 | 0.3 | 1.6 | 26.6 | 40.6 | 23.4 | 7.8 | 3.1 | 3.0 | 0.3 | 2.0 | 0.3 | NC |
| It66 | 8.3 | 15.3 | 40.3 | 23.6 | 12.5 | 3.2 | 3.0 | 0.3 | 1.0 | 0.3 | 10.4 | 13.4 | 43.3 | 23.9 | 9.0 | 3.1 | 3.0 | 0.4 | 1.0 | 0.3 | 10.9 | 17.2 | 39.1 | 25.0 | 7.8 | 3.0 | 3.0 | 0.4 | 2.0 | 0.3 | NC |
| It67 | 4.2 | 5.6 | 27.8 | 34.7 | 27.8 | 3.8 | 4.0 | 0.3 | 2.0 | 0.3 | 1.5 | 7.5 | 19.4 | 43.3 | 28.4 | 3.9 | 4.0 | 0.2 | 2.0 | 0.3 | 1.6 | 6.3 | 15.6 | 53.1 | 23.4 | 3.9 | 4.0 | 0.2 | 0.0 | 0.4 | A3-B |
| It68 | 1.4 | 4.2 | 19.4 | 43.1 | 31.9 | 4.0 | 4.0 | 0.2 | 1.3 | 0.3 | 0.0 | 0.0 | 9.0 | 53.7 | 37.3 | 4.3 | 4.0 | 0.1 | 1.0 | 0.4 | - | - | - | - | - | - | - | - | - | - | A2-B |
| It69 | 0.0 | 5.6 | 15.3 | 34.7 | 44.4 | 4.2 | 4.0 | 0.2 | 1.0 | 0.3 | 0.0 | 3.0 | 11.9 | 28.4 | 56.7 | 4.4 | 5.0 | 0.2 | 1.0 | 0.4 | - | - | - | - | - | - | - | - | - | - | A2-A |
| It70 | 0.0 | 5.6 | 11.1 | 37.5 | 45.8 | 4.2 | 4.0 | 0.2 | 1.0 | 0.4 | 0.0 | 3.0 | 9.0 | 31.3 | 56.7 | 4.4 | 5.0 | 0.2 | 1.0 | 0.4 | - | - | - | - | - | - | - | - | - | - | A2-A |
| It71 | 0.0 | 2.8 | 19.4 | 31.9 | 45.8 | 4.2 | 4.0 | 0.2 | 1.0 | 0.3 | 0.0 | 0.0 | 14.9 | 23.9 | 61.2 | 4.5 | 5.0 | 0.2 | 1.0 | 0.4 | - | - | - | - | - | - | - | - | - | - | A2-A |
| It72 | 6.9 | 8.3 | 40.3 | 30.6 | 13.9 | 3.4 | 3.0 | 0.3 | 1.0 | 0.3 | 7.5 | 10.4 | 40.3 | 31.3 | 10.4 | 3.3 | 3.0 | 0.3 | 1.0 | 0.3 | 7.8 | 14.1 | 42.2 | 29.7 | 6.3 | 3.1 | 3.0 | 0.3 | 1.0 | 0.3 | NC |
| It73 | 1.4 | 9.7 | 25.0 | 34.7 | 29.2 | 3.8 | 4.0 | 0.3 | 2.0 | 0.3 | 1.5 | 9.0 | 23.9 | 38.8 | 26.9 | 3.8 | 4.0 | 0.3 | 2.0 | 0.3 | 0.0 | 9.4 | 20.3 | 43.8 | 26.6 | 3.9 | 4.0 | 0.2 | 2.0 | 0.3 | NC |
| It74 | 8.3 | 20.8 | 31.9 | 31.9 | 6.9 | 3.1 | 3.0 | 0.3 | 2.0 | 0.2 | 13.4 | 16.4 | 35.8 | 32.8 | 1.5 | 2.9 | 3.0 | 0.4 | 2.0 | 0.3 | 12.5 | 20.3 | 37.5 | 28.1 | 1.6 | 2.9 | 3.0 | 0.4 | 2.0 | 0.3 | NC |
| It75 | 8.3 | 9.7 | 43.1 | 27.8 | 11.1 | 3.2 | 3.0 | 0.3 | 1.0 | 0.3 | 11.9 | 10.4 | 43.3 | 28.4 | 6.0 | 3.1 | 3.0 | 0.3 | 1.0 | 0.3 | 10.9 | 12.5 | 46.9 | 26.6 | 3.1 | 3.0 | 3.0 | 0.3 | 1.0 | 0.3 | NC |
| It76 | 1.4 | 2.8 | 9.7 | 44.4 | 41.7 | 4.2 | 4.0 | 0.2 | 1.0 | 0.4 | 0.0 | 4.5 | 4.5 | 49.3 | 41.8 | 4.3 | 4.0 | 0.2 | 1.0 | 0.4 | - | - | - | - | - | - | - | - | - | - | A2-B |
| It77 | 2.8 | 2.8 | 13.9 | 36.1 | 44.4 | 4.2 | 4.0 | 0.2 | 1.0 | 0.3 | 0.0 | 4.5 | 3.0 | 35.8 | 56.7 | 4.4 | 5.0 | 0.2 | 1.0 | 0.4 | - | - | - | - | - | - | - | - | - | - | A2-A |
| It78 | 2.8 | 2.8 | 13.9 | 38.9 | 41.7 | 4.1 | 4.0 | 0.2 | 1.0 | 0.3 | 7.5 | 4.5 | 4.5 | 37.3 | 46.3 | 4.1 | 4.0 | 0.3 | 1.0 | 0.4 | - | - | - | - | - | - | - | - | - | - | A2-B |
| It79 | 1.4 | 2.8 | 13.9 | 36.1 | 45.8 | 4.2 | 4.0 | 0.2 | 1.0 | 0.4 | 0.0 | 4.5 | 3.0 | 40.3 | 52.2 | 4.4 | 5.0 | 0.2 | 1.0 | 0.4 | - | - | - | - | - | - | - | - | - | - | A2-A |
| It80 | 4.2 | 13.9 | 40.3 | 25.0 | 16.7 | 3.4 | 3.0 | 0.3 | 1.0 | 0.3 | 10.4 | 13.4 | 37.3 | 25.4 | 13.4 | 3.2 | 3.0 | 0.4 | 1.0 | 0.2 | 12.5 | 12.5 | 40.6 | 25.0 | 9.4 | 3.1 | 3.0 | 0.4 | 1.3 | 0.3 | NC |
| It81 | 4.2 | 12.5 | 29.2 | 31.9 | 22.2 | 3.6 | 4.0 | 0.3 | 1.0 | 0.2 | 10.4 | 11.9 | 22.4 | 37.3 | 17.9 | 3.4 | 4.0 | 0.4 | 1.0 | 0.2 | 12.5 | 10.9 | 20.3 | 42.2 | 14.1 | 3.3 | 4.0 | 0.4 | 1.0 | 0.3 | NC |
| It82 | 4.2 | 8.3 | 27.8 | 38.9 | 20.8 | 3.6 | 4.0 | 0.3 | 1.0 | 0.3 | 3.0 | 9.0 | 26.9 | 41.8 | 19.4 | 3.7 | 4.0 | 0.3 | 1.0 | 0.3 | 6.3 | 6.3 | 20.3 | 54.7 | 12.5 | 3.6 | 4.0 | 0.3 | 1.0 | 0.4 | NC |
| It83 | 5.6 | 15.3 | 23.6 | 38.9 | 16.7 | 3.5 | 4.0 | 0.3 | 1.0 | 0.3 | 9.0 | 11.9 | 19.4 | 41.8 | 17.9 | 3.5 | 4.0 | 0.3 | 1.0 | 0.3 | 10.9 | 6.3 | 18.8 | 50.0 | 14.1 | 3.5 | 4.0 | 0.3 | 1.0 | 0.3 | NC |
| It84 | 1.4 | 6.9 | 8.3 | 38.9 | 44.4 | 4.2 | 4.0 | 0.2 | 1.0 | 0.4 | 0.0 | 4.5 | 7.5 | 40.3 | 47.8 | 4.3 | 4.0 | 0.2 | 1.0 | 0.4 | - | - | - | - | - | - | - | - | - | - | A2-B |
| It85 | 1.4 | 8.3 | 18.1 | 33.3 | 38.9 | 4.0 | 4.0 | 0.3 | 2.0 | 0.3 | 0.0 | 7.5 | 14.9 | 32.8 | 44.8 | 4.1 | 4.0 | 0.2 | 1.0 | 0.3 | - | - | - | - | - | - | - | - | - | - | A2-B |
| It86 | 2.8 | 5.6 | 13.9 | 38.9 | 38.9 | 4.1 | 4.0 | 0.2 | 1.0 | 0.3 | 0.0 | 6.0 | 11.9 | 37.3 | 44.8 | 4.2 | 4.0 | 0.2 | 1.0 | 0.3 | - | - | - | - | - | - | - | - | - | - | A2-B |
| It87 | 0.0 | 2.8 | 8.3 | 47.2 | 41.7 | 4.3 | 4.0 | 0.2 | 1.0 | 0.4 | 1.5 | 1.5 | 3.0 | 52.2 | 41.8 | 4.3 | 4.0 | 0.2 | 1.0 | 0.4 | - | - | - | - | - | - | - | - | - | - | A2-B |
| It88 | 1.4 | 6.9 | 18.1 | 40.3 | 33.3 | 4.0 | 4.0 | 0.2 | 2.0 | 0.3 | 1.5 | 10.4 | 13.4 | 46.3 | 28.4 | 3.9 | 4.0 | 0.3 | 1.5 | 0.3 | 0.0 | 9.4 | 12.5 | 57.8 | 20.3 | 3.9 | 4.0 | 0.2 | 0.0 | 0.4 | A3-B |
| It89 | 4.2 | 6.9 | 22.2 | 40.3 | 26.4 | 3.8 | 4.0 | 0.3 | 2.0 | 0.3 | 1.5 | 3.0 | 17.9 | 47.8 | 29.9 | 4.0 | 4.0 | 0.2 | 1.0 | 0.3 | - | - | - | - | - | - | - | - | - | - | A2-B |
| It90 | 0.0 | 4.2 | 13.9 | 34.7 | 47.2 | 4.3 | 4.0 | 0.2 | 1.0 | 0.4 | 1.5 | 3.0 | 11.9 | 32.8 | 50.7 | 4.3 | 5.0 | 0.2 | 1.0 | 0.4 | - | - | - | - | - | - | - | - | - | - | A2-A |
| It91 | 0.0 | 5.6 | 18.1 | 33.3 | 43.1 | 4.1 | 4.0 | 0.2 | 1.0 | 0.3 | 1.5 | 3.0 | 11.9 | 35.8 | 47.8 | 4.3 | 4.0 | 0.2 | 1.0 | 0.4 | - | - | - | - | - | - | - | - | - | - | A2-B |
| It92 | 0.0 | 9.7 | 18.1 | 43.1 | 29.2 | 3.9 | 4.0 | 0.2 | 2.0 | 0.3 | 1.5 | 4.5 | 11.9 | 50.7 | 31.3 | 4.1 | 4.0 | 0.2 | 1.0 | 0.4 | - | - | - | - | - | - | - | - | - | - | A2-B |
| It93 | 4.2 | 12.5 | 30.6 | 40.3 | 12.5 | 3.4 | 4.0 | 0.3 | 1.0 | 0.3 | 7.5 | 11.9 | 17.9 | 50.7 | 11.9 | 3.5 | 4.0 | 0.3 | 1.0 | 0.3 | 7.8 | 12.5 | 14.1 | 60.9 | 4.7 | 3.4 | 4.0 | 0.3 | 1.0 | 0.4 | NC |
| It94 | 2.8 | 4.2 | 16.7 | 41.7 | 34.7 | 4.0 | 4.0 | 0.2 | 1.0 | 0.3 | 0.0 | 6.0 | 9.0 | 40.3 | 44.8 | 4.2 | 4.0 | 0.2 | 1.0 | 0.4 | - | - | - | - | - | - | - | - | - | - | A2-B |
| It95 | 2.8 | 5.6 | 12.5 | 50.0 | 29.2 | 4.0 | 4.0 | 0.2 | 1.0 | 0.3 | 0.0 | 3.0 | 10.4 | 55.2 | 31.3 | 4.1 | 4.0 | 0.2 | 1.0 | 0.4 | - | - | - | - | - | - | - | - | - | - | A2-B |
| It96 | 1.4 | 4.2 | 12.5 | 52.8 | 29.2 | 4.0 | 4.0 | 0.2 | 1.0 | 0.4 | 3.0 | 1.5 | 10.4 | 56.7 | 28.4 | 4.1 | 4.0 | 0.2 | 1.0 | 0.4 | - | - | - | - | - | - | - | - | - | - | A2-B |
| It97 | 0.0 | 2.8 | 13.9 | 56.9 | 26.4 | 4.1 | 4.0 | 0.2 | 1.0 | 0.4 | 0.0 | 0.0 | 11.9 | 55.2 | 32.8 | 4.2 | 4.0 | 0.2 | 1.0 | 0.4 | - | - | - | - | - | - | - | - | - | - | A2-B |
| It98 | 0.0 | 1.4 | 16.7 | 48.6 | 33.3 | 4.1 | 4.0 | 0.2 | 1.0 | 0.4 | 1.5 | 0.0 | 11.9 | 46.3 | 40.3 | 4.2 | 4.0 | 0.2 | 1.0 | 0.4 | - | - | - | - | - | - | - | - | - | - | A2-B |
| It99 | 0.0 | 0.0 | 0.0 | 25.4 | 74.6 | 4.7 | 5.0 | 0.1 | 0.5 | 0.6 | - | - | - | - | - | - | - | - | - | - | - | - | - | - | - | - | - | - | - | - | A1-A |
| It100 | 1.4 | 4.2 | 23.9 | 38.0 | 32.4 | 4.0 | 4.0 | 0.2 | 2.0 | 0.3 | 1.5 | 1.5 | 22.7 | 40.9 | 33.3 | 4.0 | 4.0 | 0.2 | 1.8 | 0.3 | 0.0 | 9.4 | 12.5 | 57.8 | 20.3 | 4.1 | 4.0 | 0.2 | 1.0 | 0.4 | A3-B |
| It101 | 0.0 | 0.0 | 4.2 | 31.0 | 64.8 | 4.6 | 5.0 | 0.1 | 1.0 | 0.5 | - | - | - | - | - | - | - | - | - | - | - | - | - | - | - | - | - | - | - | - | A1-A |
| It102 | 0.0 | 2.8 | 4.2 | 18.3 | 74.6 | 4.6 | 5.0 | 0.2 | 0.5 | 0.6 | - | - | - | - | - | - | - | - | - | - | - | - | - | - | - | - | - | - | - | - | A1-A |
| It103 | 0.0 | 5.6 | 15.5 | 31.0 | 47.9 | 4.2 | 4.0 | 0.2 | 1.0 | 0.3 | 0.0 | 3.0 | 10.6 | 28.8 | 57.6 | 4.4 | 5.0 | 0.2 | 1.0 | 0.4 | - | - | - | - | - | - | - | - | - | - | A2-A |
| It104 | 1.4 | 5.6 | 18.3 | 31.0 | 43.7 | 4.1 | 4.0 | 0.2 | 1.5 | 0.3 | 0.0 | 3.0 | 12.1 | 28.8 | 56.1 | 4.4 | 5.0 | 0.2 | 1.0 | 0.4 | - | - | - | - | - | - | - | - | - | - | A2-A |
| It105 | 1.4 | 5.6 | 26.8 | 35.2 | 31.0 | 3.9 | 4.0 | 0.2 | 2.0 | 0.3 | 0.0 | 10.6 | 22.7 | 40.9 | 25.8 | 3.8 | 4.0 | 0.2 | 1.8 | 0.3 | 1.6 | 14.3 | 14.3 | 47.6 | 22.2 | 3.7 | 4.0 | 0.3 | 1.0 | 0.3 | NC |
| It106 | 0.0 | 1.4 | 9.9 | 23.9 | 64.8 | 4.5 | 5.0 | 0.2 | 1.0 | 0.5 | - | - | - | - | - | - | - | - | - | - | - | - | - | - | - | - | - | - | - | - | A1-A |
| It107 | 1.4 | 9.9 | 26.8 | 32.4 | 29.6 | 3.8 | 4.0 | 0.3 | 2.0 | 0.3 | 0.0 | 9.1 | 27.3 | 33.3 | 30.3 | 3.8 | 4.0 | 0.3 | 2.0 | 0.3 | 0.0 | 7.9 | 25.4 | 41.3 | 25.4 | 3.8 | 4.0 | 0.2 | 1.5 | 0.3 | NC |
| It108 | 0.0 | 1.4 | 14.1 | 45.1 | 39.4 | 4.2 | 4.0 | 0.2 | 1.0 | 0.4 | 0.0 | 1.5 | 13.6 | 42.4 | 42.4 | 4.3 | 4.0 | 0.2 | 1.0 | 0.4 | - | - | - | - | - | - | - | - | - | - | A2-B |
| It109 | 0.0 | 1.4 | 5.6 | 42.3 | 50.7 | 4.4 | 5.0 | 0.2 | 1.0 | 0.4 | - | - | - | - | - | - | - | - | - | - | - | - | - | - | - | - | - | - | - | - | A1-A |
| It110 | 0.0 | 9.9 | 31.0 | 38.0 | 21.1 | 3.7 | 4.0 | 0.2 | 1.0 | 0.3 | 0.0 | 4.5 | 33.3 | 39.4 | 22.7 | 3.8 | 4.0 | 0.2 | 1.0 | 0.3 | 0.0 | 9.5 | 28.6 | 39.7 | 22.2 | 3.7 | 4.0 | 0.2 | 1.0 | 0.3 | NC |
| It111 | 0.0 | 11.3 | 29.6 | 33.8 | 25.4 | 3.7 | 4.0 | 0.3 | 1.5 | 0.3 | 0.0 | 6.1 | 31.8 | 37.9 | 24.2 | 3.8 | 4.0 | 0.2 | 1.0 | 0.3 | 0.0 | 9.5 | 28.6 | 41.3 | 20.6 | 3.7 | 4.0 | 0.2 | 1.0 | 0.3 | NC |
| It112 | 0.0 | 8.5 | 28.2 | 38.0 | 25.4 | 3.8 | 4.0 | 0.2 | 1.5 | 0.3 | 0.0 | 7.6 | 27.3 | 40.9 | 24.2 | 3.8 | 4.0 | 0.2 | 1.0 | 0.3 | 0.0 | 9.5 | 19.0 | 52.4 | 19.0 | 3.8 | 4.0 | 0.2 | 1.0 | 0.3 | NC |
| It113 | 0.0 | 4.2 | 4.2 | 43.7 | 47.9 | 4.4 | 4.0 | 0.2 | 1.0 | 0.4 | 0.0 | 1.5 | 4.5 | 40.9 | 53.0 | 4.5 | 5.0 | 0.1 | 1.0 | 0.4 | - | - | - | - | - | - | - | - | - | - | A2-A |
| It114 | 1.4 | 7.0 | 15.5 | 39.4 | 36.6 | 4.0 | 4.0 | 0.2 | 1.0 | 0.3 | 1.5 | 6.1 | 10.6 | 40.9 | 40.9 | 4.1 | 4.0 | 0.2 | 1.0 | 0.3 | - | - | - | - | - | - | - | - | - | - | A2-B |
| It115 | 0.0 | 0.0 | 8.5 | 33.8 | 57.7 | 4.5 | 5.0 | 0.1 | 1.0 | 0.4 | - | - | - | - | - | - | - | - | - | - | - | - | - | - | - | - | - | - | - | - | A1-A |
| It116 | 0.0 | 0.0 | 5.6 | 38.0 | 56.3 | 4.5 | 5.0 | 0.1 | 1.0 | 0.5 | - | - | - | - | - | - | - | - | - | - | - | - | - | - | - | - | - | - | - | - | A1-A |
| It117 | 0.0 | 0.0 | 5.6 | 45.1 | 49.3 | 4.4 | 4.0 | 0.1 | 1.0 | 0.4 | 0.0 | 0.0 | 3.0 | 42.4 | 54.5 | 4.5 | 5.0 | 0.1 | 1.0 | 0.5 | - | - | - | - | - | - | - | - | - | - | A2-A |
| It118 | 0.0 | 0.0 | 4.2 | 39.4 | 56.3 | 4.5 | 5.0 | 0.1 | 1.0 | 0.5 | - | - | - | - | - | - | - | - | - | - | - | - | - | - | - | - | - | - | - | - | A1-A |
| It119 | 0.0 | 0.0 | 7.0 | 36.6 | 56.3 | 4.5 | 5.0 | 0.1 | 1.0 | 0.4 | - | - | - | - | - | - | - | - | - | - | - | - | - | - | - | - | - | - | - | - | A1-A |
| It120 | 0.0 | 0.0 | 7.0 | 42.3 | 50.7 | 4.4 | 5.0 | 0.1 | 1.0 | 0.4 | - | - | - | - | - | - | - | - | - | - | - | - | - | - | - | - | - | - | - | - | A1-A |
| It121 | 0.0 | 0.0 | 8.5 | 40.8 | 50.7 | 4.4 | 5.0 | 0.1 | 1.0 | 0.4 | - | - | - | - | - | - | - | - | - | - | - | - | - | - | - | - | - | - | - | - | A1-A |
| It122 | 0.0 | 2.8 | 7.0 | 39.4 | 50.7 | 4.4 | 5.0 | 0.2 | 1.0 | 0.4 | - | - | - | - | - | - | - | - | - | - | - | - | - | - | - | - | - | - | - | - | A1-A |
| It123 | 0.0 | 5.6 | 11.3 | 39.4 | 43.7 | 4.2 | 4.0 | 0.2 | 1.0 | 0.4 | 1.5 | 6.1 | 4.5 | 39.4 | 48.5 | 4.3 | 4.0 | 0.2 | 1.0 | 0.4 | - | - | - | - | - | - | - | - | - | - | A2-B |
| It124 | 1.4 | 2.8 | 11.3 | 39.4 | 45.1 | 4.2 | 4.0 | 0.2 | 1.0 | 0.4 | 0.0 | 3.0 | 6.1 | 37.9 | 53.0 | 4.4 | 5.0 | 0.2 | 1.0 | 0.4 | - | - | - | - | - | - | - | - | - | - | A2-A |
| It125 | 0.0 | 4.2 | 19.7 | 36.6 | 39.4 | 4.1 | 4.0 | 0.2 | 1.0 | 0.3 | 0.0 | 7.6 | 13.6 | 31.8 | 47.0 | 4.2 | 4.0 | 0.2 | 1.0 | 0.3 | - | - | - | - | - | - | - | - | - | - | A2-B |
| It126 | 1.4 | 4.2 | 21.1 | 36.6 | 36.6 | 4.0 | 4.0 | 0.2 | 2.0 | 0.3 | 0.0 | 13.6 | 15.2 | 34.8 | 36.4 | 3.9 | 4.0 | 0.3 | 2.0 | 0.3 | 0.0 | 22.2 | 11.1 | 34.9 | 31.7 | 3.8 | 4.0 | 0.3 | 2.0 | 0.3 | NC |
| It127 | 1.4 | 5.6 | 23.9 | 47.9 | 21.1 | 3.8 | 4.0 | 0.2 | 1.0 | 0.3 | 1.5 | 6.1 | 16.7 | 47.0 | 28.8 | 4.0 | 4.0 | 0.2 | 1.0 | 0.3 | - | - | - | - | - | - | - | - | - | - | A2-B |
| It128 | 1.4 | 1.4 | 12.7 | 35.2 | 49.3 | 4.3 | 4.0 | 0.2 | 1.0 | 0.4 | 1.5 | 1.5 | 9.1 | 24.2 | 63.6 | 4.5 | 5.0 | 0.2 | 1.0 | 0.5 | - | - | - | - | - | - | - | - | - | - | A2-A |
| It129 | 1.4 | 7.0 | 15.5 | 43.7 | 32.4 | 4.0 | 4.0 | 0.2 | 1.0 | 0.3 | 0.0 | 6.1 | 12.1 | 42.4 | 39.4 | 4.2 | 4.0 | 0.2 | 1.0 | 0.3 | - | - | - | - | - | - | - | - | - | - | A2-B |
| It130 | 1.4 | 7.0 | 21.1 | 36.6 | 33.8 | 3.9 | 4.0 | 0.2 | 2.0 | 0.3 | 0.0 | 6.1 | 15.2 | 37.9 | 40.9 | 4.1 | 4.0 | 0.2 | 1.0 | 0.3 | - | - | - | - | - | - | - | - | - | - | A2-B |

SD: Strongly disagree; D: Disagree; NAD: Neither agree nor disagree; A: Agree; SA: Strongly agree; M: Mean; CV: Coefficient of Variation; Me: Median; IQR: Interquartile range; Pi: Scott's Pi inter-rater reliability coefficient.

A1-A: Approved in Round 1, by Absolute Majority (SA =>50% and SD+D <=33.3%)

A2-A: Approved in Round 2, by Absolute Majority (SA =>50% and SD+D <=33.3%)

A2-B: Approved in Round 2, by Qualified Majority (SA+A =>75%)

A3-B: Approved in Round 3, by Qualified Majority (SA+A =>75%)

R1-A: Rejected in Round 1, by Absolute Majority (SD+D =>50%)

NC: No consensus
